# Polycomb Protein SCML2 Regulates the Cell Cycle by Binding and Modulating CDK/CYCLIN/p21 Complexes

**DOI:** 10.1371/journal.pbio.1001737

**Published:** 2013-12-17

**Authors:** Emilio Lecona, Luis Alejandro Rojas, Roberto Bonasio, Andrew Johnston, Oscar Fernández-Capetillo, Danny Reinberg

**Affiliations:** 1 Howard Hughes Medical Institute, Department of Biochemistry, New York University School of Medicine, New York, New York, United States of America; 2 Spanish National Cancer Research Centre (CNIO), Molecular Oncology Programme, Madrid, Spain; Harvard University, United States of America

## Abstract

Polycomb group (PcG) proteins are transcriptional repressors of genes involved in development and differentiation, and also maintain repression of key genes involved in the cell cycle, indirectly regulating cell proliferation. The human *SCML2* gene, a mammalian homologue of the *Drosophila* PcG protein SCM, encodes two protein isoforms: SCML2A that is bound to chromatin and SCML2B that is predominantly nucleoplasmic. Here, we purified SCML2B and found that it forms a stable complex with CDK/CYCLIN/p21 and p27, enhancing the inhibitory effect of p21/p27. SCML2B participates in the G1/S checkpoint by stabilizing p21 and favoring its interaction with CDK2/CYCE, resulting in decreased kinase activity and inhibited progression through G1. In turn, CDK/CYCLIN complexes phosphorylate SCML2, and the interaction of SCML2B with CDK2 is regulated through the cell cycle. These findings highlight a direct crosstalk between the *Polycomb* system of cellular memory and the cell-cycle machinery in mammals.

## Abstract 2

A transcription-independent role is identified for the human Polycomb group protein SCML2B in regulating the cell cycle. SCML2B cooperates with p21 to inhibit CDK2/CYCE complexes during early G1, resulting in delayed entry into S phase.

## Author Summary

The processes of development and differentiation require an exquisite coordination of the gene expression program with the proliferation of the cells. The Polycomb group of proteins are important development regulators and most research to date has focused on their involvement in maintaining epigenetic silencing of genes during development and the self-renewal and differentiation of stem cells. Up to now, we've seen that Polycomb proteins influence the transcriptional status of cell-cycle regulators via chromatin modifications. Here we describe a transcription-independent function for a human Polycomb group protein in regulating the cell cycle. We show that the Polycomb group protein SCML2 directly regulates the progression of cells from G1 into S phase by cooperating with p21 to restrain the activation of CDK2/CYCE complexes in early G1. This function is carried out by the “B” isoform of SCML2 that does not interact with the Polycomb complex PRC1. Further, SCML2B phosphorylation is regulated through the cell cycle and is partly dependent on CDK1 and CDK2. These findings highlight a direct crosstalk between the Polycomb system of cellular memory and cell-cycle machinery in mammals, providing insight into novel functions of the mammalian Polycomb system.

## Introduction

*Polycomb* group (PcG) proteins are key developmental regulators that maintain epigenetic silencing of genes [1] and determine the expression patterns of homeobox genes during embryonic development. In *Drosophila* five different PcG complexes have been described: Polycomb Repressive Complex 1 (PRC1) and 2 (PRC2) [1], Pho Repressive Complex (PhoRC) [2], Polycomb repressive deubiquitinase (PR-DUB) [3], and dRING associated factors (dRAF) [4]. PRC2 methylates lysine 27 of histone H3 (H3K27) [5],[6], whereas PRC1 compacts chromatin [7], and catalyzes the deposition of ubiquitination at H2AK119 [8], contributing to the establishment of a chromatin environment that is repressive for transcription. PRC1- and PRC2-mediated repression in *Drosophila* is partially dependent on the presence of PhoRC [9].

Research on PcG function has mostly focused on components of the PRCs and their role in transcriptional repression. However, mutations in several other PcG genes display strong homeotic phenotypes in *Drosophila*, and the products of these genes are likely to have important roles in gene regulation and epigenetic memory. One of these less studied proteins is the product of the *Sex comb on midleg* (*scm*) gene. SCM is required for the recruitment and repressive function of PRC1 and PRC2 [9], and interacts with SFMBT, a component of PhoRC [10]. SCM contains two malignant brain tumor (MBT) repeats [11], a domain of unknown function (DUF3588), an SPM/SAM domain, and two zinc fingers [12]. It associates in substoichiometric amounts with the PRC1 complex [13], likely through the interaction of its SPM domain with that of polyhomeotic (PH) [14]. However, the absence of PRC1 does not affect SCM localization to target genes, suggesting that SCM may function upstream (or independently) of PRC1–2 [9].

Like other PcG proteins, SCM exerts a repressive effect on target genes, which requires both its MBT and SPM domains [15],[16]. MBT domains bind preferentially to mono- and di-methylated lysine residues in histone tails, which might facilitate their recruitment to chromatin [17],[18]. Despite its biochemical and genetic connections with PcG complexes, very little is known about the cellular function(s) of SCM, especially in humans. There are four homologues of SCM in mammals: SCMH1 and SCML2 comprise the MBT repeats, the DUF3588 domain, and the SPM domain, whereas SCML1 only presents the C-terminal SPM domain and SCML4 comprises the DUF3588 and SPM domains [19]–[22]. Similar to *Drosophila* SCM, SCMH1 is a substoichiometric component of PRC1 [23], interacts with homologues of PH [22], and its hypomorphic mutation in mice results in homeotic transformations, defective spermatogenesis, and premature senescence of embryonic fibroblasts [24]. Other studies have suggested a role for SCMH1 and PRC1 in geminin ubiquitination, and showed that SCMH1 itself is ubiquitinated [25]. The *SCML2* gene is deleted in a subset of medulloblastomas [26], suggesting a role in tumor suppression.

In addition to the regulation of developmental genes, PcG proteins impinge on other cellular functions, such as the cell cycle or the DNA damage response [27]. Both PRC1 and PRC2 repress the *Ink4a/Arf * locus [28], restricting the expression of p16^INK4a^. This is a member of the INK4 family of proteins, which blocks CDK4 and CDK6 by inhibiting the interaction with their cyclin partner. Another family of inhibitors, the Kip family, establishes a ternary complex with the CDK/Cyclin, locking it in an inactive conformation. The regulation of these inhibitors occurs at both the transcriptional and protein level. Several mechanisms are responsible for the degradation of p21 or p27 at different phases of the cell cycle [29], modulating their stability and their inhibitory actions. Interestingly, PRC1 has been recently shown to directly regulate the stability of geminin, Mdm2, and p53 [25],[30],[31]. The regulation of these proteins can indirectly impact on cell-cycle progression and on the levels of CDK inhibitors, suggesting that the functions of PcG are not restricted to transcriptional regulation. This idea is further supported by the recent report of the direct regulation of CYCB by PRC1 components in *Drosophila* [32]. However, an analogous role in vertebrates remains unexplored.

Here we uncover a new function for SCML2B, one of the isoforms of SCML2, in the regulation of the cell cycle. Our results show that SCML2B contributes to the formation of repressive CDK2/CYCE/p21 complexes and stabilization of p21 in early G1, leading to reduced kinase activity and controlling the progression through the G1/S border. SCML2 is itself differentially phosphorylated by CDK2 and CDK1 during the cell cycle, suggesting further levels of crosstalk regulation. These findings reveal a role of a PcG protein in modulating the cell-cycle machinery in mammals.

## Results

**Figure 1:**
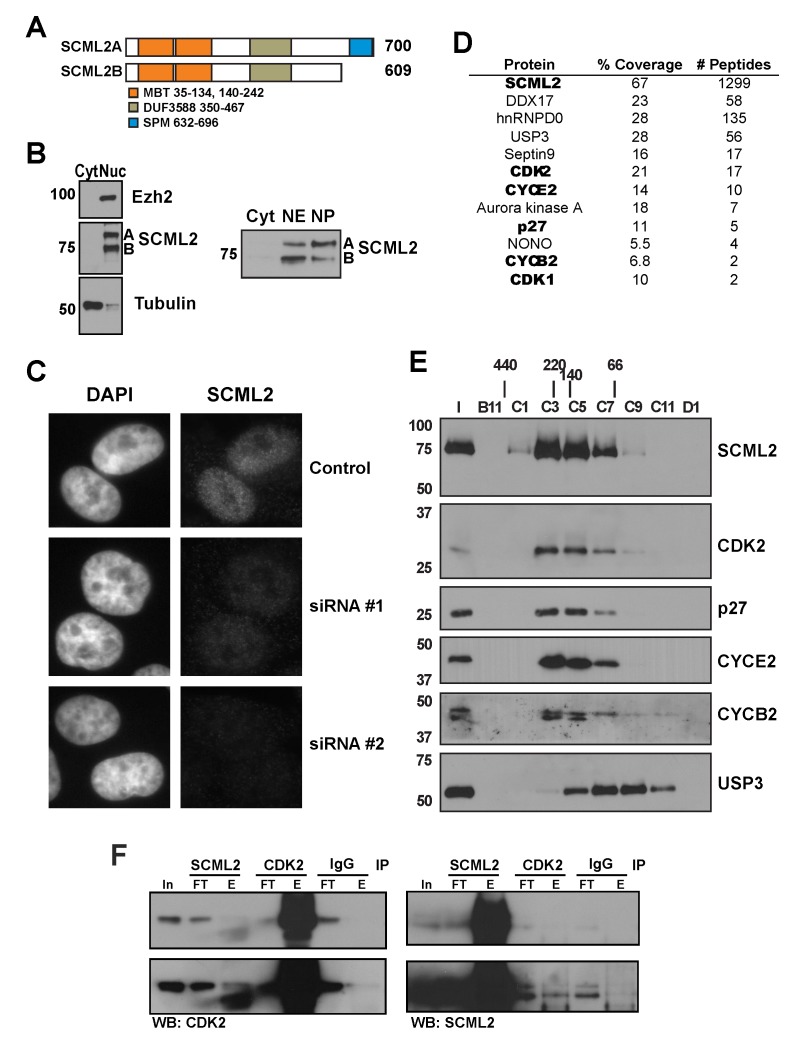
Cellular distribution and composition of SCML2B-containing complexes. (A) Schematic representation of the two protein isoforms encoded by the *SCML2* gene in *Homo sapiens*. (B) Western blot analysis of nuclear and cytoplasmic extracts of K562 cells using antibodies specific for SCML2, EZH2, and tubulin (left). Western blot of cytoplasmic, nuclear extract, and solubilized nuclear pellet of HeLa cells using an antibody specific for SCML2 (right). (C) Immunofluorescence analysis of SCML2 in HeLa cells transfected with two different siRNAs specific for SCML2. DAPI staining is shown on the left, and staining with an SCML2-specific antibody is shown on the right. (D) Proteins identified by mass spectrometry in the fraction containing purified SCML2B, indicating number of peptides for each protein and coverage. (E) Fractionation of purified SCML2B was performed by size exclusion chromatography and analyzed by Western blot for SCML2, CYCE2, CYCB2, CDK2, p27, and USP3. Molecular weight markers are indicated on the left. (F) Immunoprecipitation of CDK2 and SCML2 from nuclear extracts from HeLa cells. Five percent of the input (In) is shown, along with the flow-through (FT) and elution (E) fractions. A nonspecific IgG pull-down is shown as control.

Given that SCML2 likely performs functions independently of PRC1 [23], we sought to elucidate its PRC1-independent role(s). RT-PCR for *SCML2* uncovered two different RNA species, one of them encoding the full-length protein (SCML2A) and another lacking the region encoding the SPM domain (SCML2B) (shown schematically in Figure 1A). Interestingly, the SPM domain of SCM is required for its repressive function and, when expressed alone, acts as a dominant negative in *Drosophila* [16]. These data raised the possibility that the SPM domain is important for the localization of SCM to chromatin and that SCML2A and SCML2B may have different subcellular distributions. We generated an antibody against the central region of SCML2 and confirmed that both SCML2A and SCML2B protein species are expressed in several different cell lines (Figure 1B and Figure S1A–C). Both isoforms were recovered in the nuclear fraction of K562 cell extracts, irrespective of the presence of the SPM domain (Figure 1B, left). We further fractionated the nuclear compartment separating proteins extracted with 400 mM NaCl (nucleoplasmic fraction) from those tightly bound to the chromatin, which cannot be solubilized under these conditions (chromatin fraction). SCML2B was predominantly found in the nucleoplasm, whereas SCML2A associated more tightly to chromatin (Figure 1B, right and Figure S1A and B). The nuclear localization of SCML2 was confirmed by immunofluorescence in HeLa cells (Figure 1C). Our antibody showed no reactivity in HeLa cells treated with siRNAs designed to target simultaneously *SCML2A* and *SCML2B* (Figure 1C, bottom panels), confirming its specificity. An antibody raised against a region only present in SCML2A only recognized this isoform, further confirming the identity of the bands detected with the SCML2 antibody (Figure S1C).

### Purification of Endogenous SCML2B

We purified SCML2 from nuclear extract of HeLa-S3 cells using conventional chromatography (Figure S1D). SCML2B is the most abundant isoform present in this fraction, and during the purification, SCML2A and SCML2B separated across the MonoS column, indicating that they form distinct complexes. The putative SCML2B-containing complex eluted with an apparent molecular weight of 150–200 kDa during size exclusion chromatography. Polypeptides co-eluting with SCML2B in the final chromatographic step were identified by mass spectrometry of the fraction showing the peak of SCML2 signal (Figure 1D and Figure S1E, arrow). SCML2B was the most abundant protein identified, and the absence of peptides mapping to the SPM domain (which is exclusive to SCML2A) confirmed that SCML2B is part of at least one complex that does not contain SCML2A. In addition, we detected two cyclins (CYCE2 and CYCB2) and two CDKs (CDK1 and CDK2), along with the CDK/CYC inhibitor p27 (Figure 1E). We excised different regions containing the bands that show a similar elution profile to SCML2B, and the results from mass spectrometry indicated that CYCE2, CYCB2, CDK1, CDK2, and p27 could indeed be forming a complex with SCML2B (Figure S1E and Tables S1, S2, S3, S4). In order to confirm whether these proteins form a stable complex with SCML2B, we subjected the material from this step of purification (hydroxyapatite chromatography, Figure S1D) to size exclusion chromatography and found that CDK2, CYCE2, CYCB2, and p27 co-eluted with SCML2B (Figure 1E). The apparent molecular weight of this complex was 170 kDa, in agreement with the sum of the sizes of CDK2, CYCE2, p27, and SCML2B. In contrast, although USP3 and DDX17 were detected in the purified material by mass spectrometry (Figure 1D), they either eluted as a monomer from the size exclusion column (Figure 1E, USP3) or were not detectable in the co-elution (DDX17), suggesting that they do not form a stable complex with SCML2B. We confirmed that SCML2 and CDK2 interact by reciprocal immunoprecipitation in nuclear extract from HeLa cells (Figure 1F). The antibody for SCML2 is targeted to a region between the DUF and the SPM domains. As this region mediates the interaction with CDK2/CYCE complexes (see below), the pull-down of SCML2 may partially disrupt the interaction with CDK2, explaining why this immunoprecipitation is not very efficient. The interaction can also be detected in nuclear extracts from HCT116 cells (Figure S1F) and other cell types (unpublished data). The potential interaction with other proteins present in this fraction was tested by immunoprecipitation from nuclear extracts, and we failed to detect an interaction with Septin-9, hnRNPD0, or NONO (unpublished data). Thus, our biochemical purification uncovered the existence of a complex between the PcG protein SCML2B and a core component of the cell-cycle machinery, the CDK/CYC/p27 complex.

### SCML2B Associates with CDK/CYC/p21-p27 *in Vitro*

We next analyzed the interaction of SCML2B with CDK/CYC complexes and p27 *in vitro* employing recombinant proteins purified from either bacteria (His-SCML2B, and p27) or insect cells (HA-CDK2/His-CYCE, HA-CDK2/His-CYCA, and CDK1/His-CYCB) (Figure S2A–C). Recombinant SCML2B interacted with the CDK2/CYCE complex (Figure S2D, left) and with p27 (Figure S2D, middle) separately, and the interaction with a preformed CDK2/CYCE/p27 complex was stronger (Figure S2D, right). SCML2B also interacted with CDK2/CYCA and CDK1/CYCB (Figure S2E and F) as well as with p21, either alone or in a complex with CDK2/CYCE (Figure S3B), suggesting a role for SCML2B in the function of these cell-cycle regulators. We mapped the domains in SCML2B responsible for the interaction employing the different fragments of SCML2B depicted in Figure S3A. GST-p21 pull-down indicated that two regions in SCML2B mediate interactions with p21 or the CDK2/CYCE/p21 complex: the MBT repeats at the N-terminus of the protein, and a region between the DUF3588 and the SPM domains, predicted to be unstructured (Ran) (Figure S3C). The MBT-DUF fragment was more efficiently pulled down by GST-p21 than the MBT repeats alone, indicating that the DUF domain may be important to structurally favor the interaction (Figure S3C).

### SCML2B Stimulates p21– and p27–CDK/CYC Interactions

The results thus far suggested that SCML2B might bind to p21/p27 and CDK/CYC complexes in a cooperative manner. Indeed, substoichiometric and stoichiometric amounts of p21 stimulated the interaction between SCML2B and CDK2/CYCE in a dose-dependent manner (Figure S3D, lanes 2 and 3), but excess p21 had the opposite effect (Figure S3D, lane 4). Several sites of interaction have been described between CDK2/CYCE and p21, and it has been postulated that more than one p21 or p27 molecule can bind to the CDK2/CYCE complex to achieve full repression [33], although one molecule of p21 is sufficient to repress CDK/CYCE complexes [34], in line with the crystal structure of the CDK2/CYCA complex in the presence of the inhibitory domain of p27 [35]. We cannot rule out that additional binding surfaces are present in regions of p27 outside of the inhibitory domain, but our results indicate that excess p21 blocks the binding sites of CDK2/CYCE within SCML2B. The incubation of CDK2/CYCE with increasing amounts of SCML2B also resulted in the stimulation of the interaction with GST-p21 (Figure S3E). A similar effect was detected when the complexes were pulled down by GST-p21 (Figure S3F, compare lanes 1–2 and 5–6).

To further confirm that p21/p27 and SCML2B bind cooperatively to CDK/CYC complexes, we reconstituted the complex stepwise with recombinant proteins and subjected it to size exclusion chromatography. Only a small part of CDK2 co-eluted with SCML2B in the absence of p27 (Figure S4, left), indicating that the interaction with CDK2/CYCE alone is weak. The addition of p27 resulted in a change in the migration of both SCML2B and CDK2 that now co-eluted with p27 (Figure S4, right), further supporting that the binding of SCML2B to CDK complexes is stimulated by p21/p27.

In summary, our *in vitro* experiments show that SCML2B directly interacts with CDK2/CYCE complexes and that the presence of p21/p27 is required to stabilize the interaction.

### SCML2B Inhibits CDK2/CYCE Enzymatic Activity

Next, we sought to determine the functional consequences of SCML2B association with CDK/CYC/p21-p27. We monitored CDK/CYC kinase activity towards histone H1e *in vitro*, following its phosphorylation at residue T146 with a phospho-specific antibody (Figure S5A). The analysis of the phosphorylation of a single residue allows the determination of the kinetic parameters of a single reaction, avoiding measuring different events at the same time. In this way we avoid confounding effects due to a mixture of reactions with different parameters being measured in the same experiment. All the recombinant CDK/CYC complexes tested were active towards H1eT146, and in each case, H1e phosphorylation was inhibited by the addition of increasing amounts of p27, as expected (Figure S5B). We then compared the kinase activity of CDK2/CYCE when associated with either SCML2B or p27 or both, using reconstituted complexes that were fractionated by size exclusion chromatography (Figure S4). The presence of SCML2B had no effect on the activity of CDK2/CYCE in the absence of p27, but resulted in a significant inhibition of p27-containing CDK2/CYCE complexes (Figure S5C–E), even considering that p27 alone reduced the activity of CDK2/CYCE (Figure S5C, compare bar 1 with 4).

**Figure 2:**
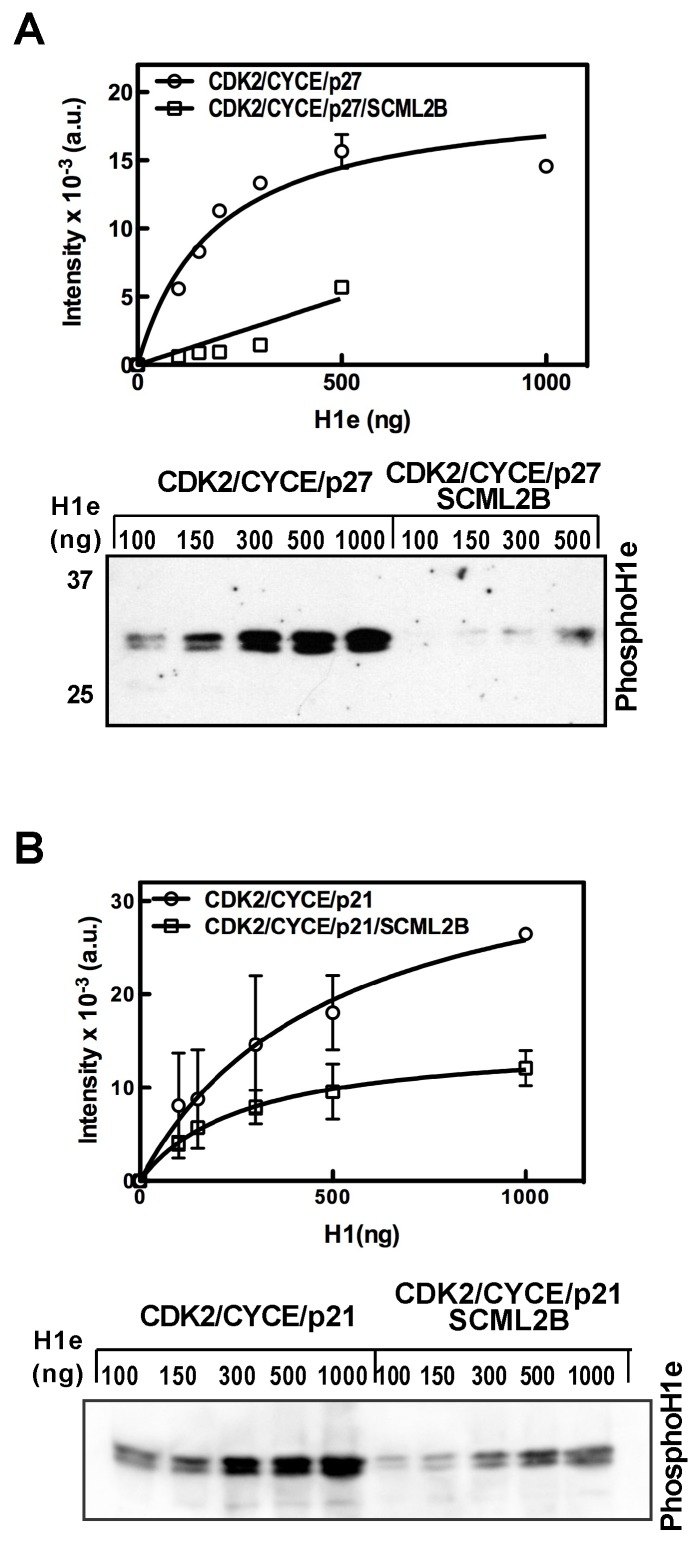
Effect of SCML2B on the activity of CDK2/CYCE/p21-p27 *in vitro*. (A–B) Kinetic analysis of the kinase activity of CDK2/CYCE/p27 or CDK2/CYCE/p27/SCML2B (A) and CDK2/CYCE/p21 or CDK2/CYCE/p21/SCML2B (B) complexes with increasing concentrations of histone H1e, with the products analyzed by Western blot using antibody specific to H1T146Ph (lower panel) and quantified by densitometric analysis of the bands from three different experiments (upper panel). The results were fitted to Michaelis–Menten kinetics, and the amount of histone H1e was in 2–20 molar excess of SCML2B.

A detailed analysis of H1e phosphorylation using a mixture of CDK2/CYCE and p27 showed that the activity of this complex follows Michaelis-Menten kinetics and that the presence of SCML2B almost abolished the residual activity of the CDK2/CYCE/p27 complex (Figure 2A). This effect was not limited to p27 as SCML2B also enhanced the inhibitory effect of p21 on CDK2/CYCE kinase activity (Figure 2B). These results, together with the *in vitro* interaction experiments, suggest that SCML2B has an inhibitory effect on CDK/CYC through the stabilization of their interaction with p27 and p21. The addition of SCML2B reduced the V_max_ of the reaction without significantly changing the affinity for H1e, indicating that even if SCML2B is itself a substrate of CDK2 (see below), it is not competing out H1e under the reaction conditions, where H1e is present at ≥2-fold molar excess versus SCML2B.

### Expression and Phosphorylation of SCML2 Through the Cell Cycle

**Figure 3:**
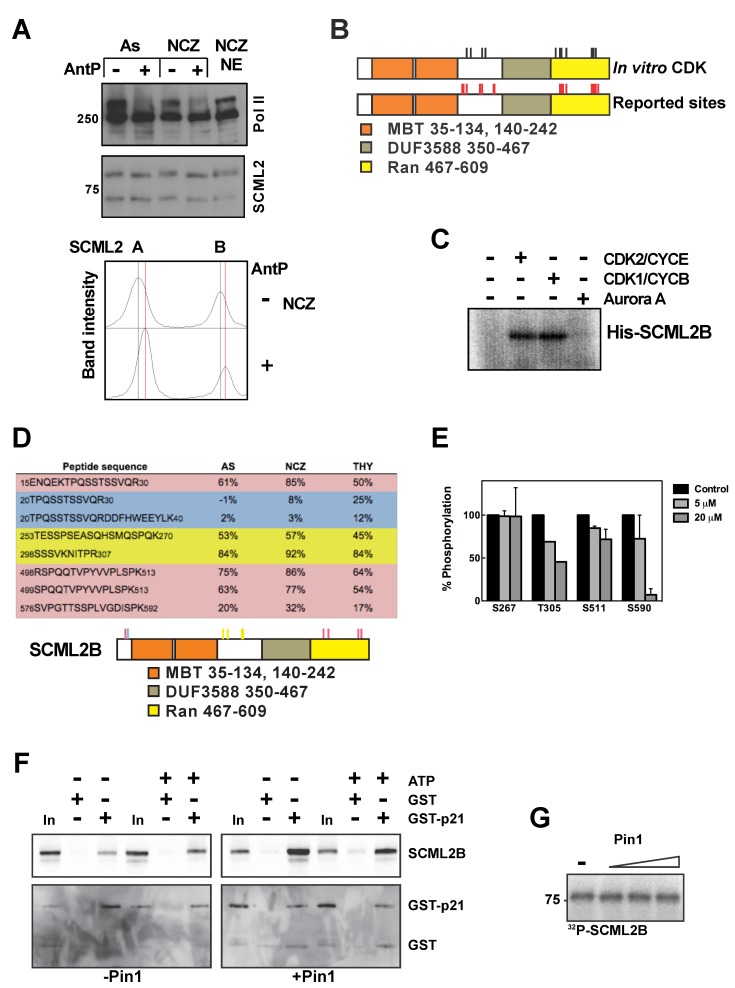
Expression and phosphorylation of SCML2 through the cell cycle. (A) Western blot analysis of nuclear extracts derived from HeLa cells growing asynchronously or arrested in mitosis with nocodazole, and incubated in the absence (−) or presence (+) of Antarctic phosphatase (top panel). The blot was probed for SCML2, and RNA polymerase II was used as a control. A densitometric analysis of the results is shown, with the positions of the peaks without phosphatase treatment indicated by a black line, and those derived from Antarctic phosphatase treatment with a red line (bottom panel). (B) Schematic representation of all the phospho-residues identified in the mass spectrometry analysis of SCML2B phosphorylated by CDK2/CYCE or CDK1/CYCB (black sticks). The phospho-sites of SCML2B previously identified in two phospho-proteomics reports are also shown (red sticks). (C) Autoradiography of SCML2B phosphorylated *in vitro* with CDK2/CYCE, CDK1/CYCB, Aurora kinase A, or in the absence of kinase, employing ^32^P-γATP. (D) Quantification of the phosphorylation of SCML2B peptides from nuclear extracts of 293 cells growing asynchronously (AS) or arrested in mitosis with nocodazole (NCZ) and in S phase with thymidine (THY). Peptides with a higher level of phosphorylation in mitosis are highlighted in red, those with a higher level of phosphorylation in S phase are highlighted in blue, and those with a constant level of phosphorylation are highlighted in yellow. A schematic of SCML2B showing the phosphorylated residues is shown below. (E) Quantification of the phosphorylation of SCML2B peptides from nuclear extracts of 293 cells treated with increasing concentrations of Roscovitine for 8 h. (F) SCML2B was phosphorylated with CDK2/CYCE complexes in the absence (left panel) or presence (right panel) of Pin1, and the reaction was carried out with or without ATP, as indicated on top. After removing the CDK2/CYCE complexes in Ni-NTA column, SCML2B was incubated with GST alone or GST-p21. The proteins were pulled down using Glutathione sepharose beads. Five percent of the input is shown (In) as control. The pull-downs were analyzed by Western blot using specific antibodies for SCML2, GST, or CDK2, as indicated. (G) Autoradiography of SCML2B phosphorylated *in vitro* with CDK2/CYCE in the presence of increasing concentrations of Pin1 or without Pin1 (−), employing ^32^P-γATP.

As SCML2B interacts *in vivo* (Figure 1), and *in vitro* (Figures S2 and S3), with CDK/CYC complexes that function during different phases of the cell cycle, we next tested whether SCML2B expression is itself subjected to cell-cycle regulation. After release from a double thymidine block (Figure S6A), SCML2A and SCML2B protein levels showed a similar profile, with small fluctuations during the cell cycle in HeLa and U2OS cells (Figure S6B and unpublished data). Both SCML2 isoforms were expressed at higher levels in S compared to early G1 and G2/M. SCML2 exhibited slightly altered gel mobility in G2/M (see below), suggesting cell-cycle–dependent posttranslational modification(s) of the protein. We compared the mobility of SCML2 in extracts obtained from asynchronous HeLa cells (As), or HeLa cells arrested in G0 by serum starvation (SS), G1/S by double thymidine block (Thy), or mitosis with nocodazole (NCZ), and found that the mobility of either SCML2A or SCML2B was slowest in the case of extracts prepared from cells arrested in mitosis (Figure S6C). The mobility of each of the SCML2 isoforms increased upon treating the extracts with Antarctic phosphatase (Figure 3A), suggesting that SCML2A and SCML2B are phosphorylated during mitosis. Consistent with this, two proteomic studies reported that SCML2 is phosphorylated at several Ser and Thr residues (shown schematically in Figure 3B) in cells arrested in mitosis after nocodazole treatment [36],[37].

These results indicate that SCML2 might itself be regulated through its phosphorylation mediated by its interaction partners, CDK/CYC. We confirmed that both CDK2/CYCE and CDK1/CYCB phosphorylate SCML2B *in vitro*, while Aurora kinase A was ineffectual (Figure 3C). Mass spectrometry of the products of *in vitro* phosphorylation reactions revealed that CDK2/CYCE and CDK1/CYCB targeted similar sites on SCML2B (Table S5 and Figure S7A), some of which are preferentially phosphorylated during mitosis *in vivo* (Table S5) [36],[37]. Analysis of their sequence using the Phosida software [38] revealed that S267 and T305 are embedded within a consensus CDK2 and CDK1 target, and S511 and S590 are in the context of a CDK1 motif. The target sites of CDK phosphorylation were concentrated between the MBT repeats and the DUF3588 domain and in two Ser/Thr-rich stretches in the Ran region, which is important for the interaction with CDK/CYC/p21-p27 complexes (Figure 3B).

Next, we analyzed the phosphorylation status of SCML2B during different phases of the cell cycle *in vivo*. To this end, we induced expression of transgenic Flag-One-STrEP-tagged SCML2B (FS-SCML2B) in stably transfected 293T-REx cells, and analyzed its phosphorylation status by mass spectrometry after arresting the cells in mitosis or S phase (Figure S7B). By comparing the signal from *in vitro* dephosphorylated and untreated peptides, we observed that SCML2B was preferentially phosphorylated in mitosis at several residues (Figure 3D, red), including some of the *in vitro* CDK/CYC targets (S499 and S511) (Table S5). In contrast, other residues displayed a similar level of phosphorylation in mitosis and S phase (Figure 3D, yellow), while the phosphorylation in a Ser/Thr stretch in the N-terminal region was higher in S phase (Figure 3D, blue). We confirmed that T305, S511, and S590 are phosphorylated by CDK1/2 *in vivo*, as treatment of cells with increasing concentrations of Roscovitine (an inhibitor for CDK1 and CDK2) for 8 h reduced the levels of phosphorylation of these residues (Figure 3E). In contrast, phosphorylation of S267 was not affected by the inhibition of CDK1/2. The treatment with Roscovitine did not induce major changes in the cell-cycle distribution of the cells (Figure S7C). These data confirmed that SCML2B is highly phosphorylated during mitosis and that this phosphorylation is partly mediated by CDK1/2.

We analyzed the effect of the phosphorylation of SCML2 on its interaction with p21 *in vitro*. The pull-down of SCML2B by GST-p21 was not changed when SCML2B was previously phosphorylated by CDK2/CYCE (Figure 3F, left). CDK1 and CDK2 target S/T-P motifs that then become substrates for isomerization of the Pro by Pin1 [39]. Several of the residues of SCML2 phosphorylated by CDK in cells and *in vitro* are adjacent to Pro (T305, S511, S590) and reside within flexible regions that mediate the interaction with CDK2/CYCE and p21/p27 (Figure S3). The addition of Pin1 to the kinase reaction did not change the levels of phosphorylation of SCML2 by CDK2/CYCE (Figure 3G), as has been described for other substrates of Pin1 [40]. In contrast, the presence of Pin1 partially impairs the interaction of phosphorylated SCML2 with p21 (Figure 3F). These results suggest that Pin1 recognizes the phosphorylated residues in SCML2, inducing a conformational change that reduces the binding to p21, potentially restricting the actions of SCML2 during the cell cycle.

### SCML2B Regulates G1 Progression

**Figure 4:**
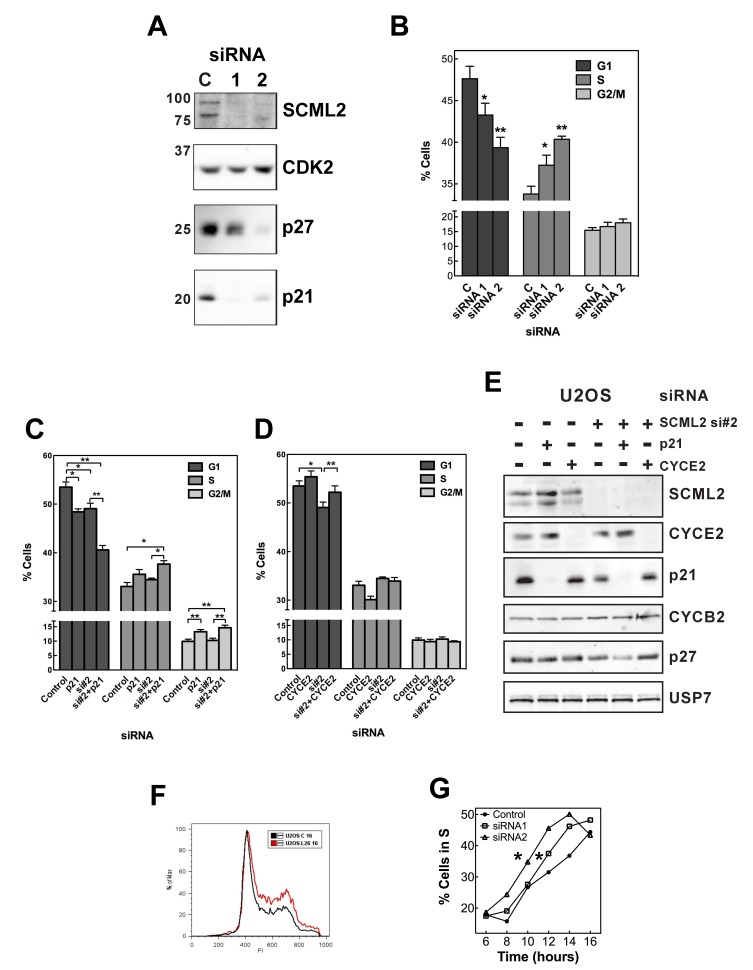
Role of SCML2 in cell cycle. (A) Western blot analysis of extracts prepared from U2OS cells treated with two different siRNAs (#1, #2) against SCML2 or a control siRNA (C), using antibodies specific to SCML2, CDK2, p27, and p21. (B) Cell-cycle distribution of U2OS cells after SCML2 knockdown using the siRNAs indicated, measured by PI staining and FACS. *p<0.05, **p<0.01. (C–D) Cell-cycle distribution of U2OS cells after treatment with a control siRNA (Control), siRNA against either SCML2 (#2) or p21, or a combination thereof for SCML2 and p21 (C), or for SCML2 (#2) and a combination of SCML2 and CYCE2 (D), measured by PI staining and FACS. Statistical significance was determined with paired *t* tests. *p<0.05, **p<0.01, ***p<0.001. (E) Western blot analysis of extracts prepared from U2OS cells treated with siRNA against either SCML2 (#2), or p21 or CYCE2, or a combination thereof for SCML2 and p21, or for SCML2 and CYCE2, or a control siRNA (first column), using antibodies specific to SCML2, CYCE2, p21, CYCB2, p27, and USP7 (loading control). (F) U2OS cells were synchronized in mitosis with nocodazole after SCML2 knockdown, and the cell-cycle distribution after 16 h of release is compared. (G) Quantification of the proportion of cells in S phase at different time points after release from mitotic arrest from the experiment shown in Figure S8. *p<0.05.

Both p21 and p27 regulate cell-cycle progression into S phase, and p21 is also involved in the transition to mitosis [29]. Phosphorylation of p21 and p27 by CDK2/CYCE complexes is required for their proteasome-mediated degradation that, in turn, allows cells to progress from G1 to S phase. Because our biochemical data showed that SCML2B interacts with CDK/CYC/p21-p27 and enhances the inhibitory effect of p21 or p27 on the kinase activity, and is itself subjected to phosphorylation as a function of the cell cycle, we reasoned that SCML2B could regulate the cell cycle *in vivo*. We transiently transfected U2OS cells with a control siRNA or two different siRNAs specific for SCML2: siRNA#1 and siRNA#2 (Figure 4A). Immunofluorescence analysis indicated that siRNA#2 was more effective than siRNA#1 (Figure 1C). None of the siRNAs affected the expression of CDK2, but knockdown of SCML2 elicited a variable decrease in the levels of p27 and a strong reduction in the levels of p21 (Figure 4A). As the knockdown of SCML2 consistently destabilized p21, we decided to focus on the role of this inhibitor, although we do not rule out a potential contribution of p27 to the functions of SCML2. Consistent with SCML2 having an impact on cell-cycle progression, knockdown of both SCML2A and SCML2B led to a decreased proportion of cells in G1 phase (5%–10%), which coincided with an increased proportion of cells in S phase and, to a lesser extent, in G2/M (Figure 4B). Overexpression of CYCE elicits a decrease in G1 phase of around 10%–20% in different cell types [41],[42], indicating that the changes observed upon SCML2 knockdown are highly significant. There was no detectable increase in cellular apoptosis upon treatment with any of the siRNAs (unpublished data).

To verify if the effects of SCML2 on the cell cycle were mediated by regulation of p21 and CDK/CYC complexes as suggested by our biochemical experiments above, we performed double knockdown experiments. Knockdown of p21 elicited a decrease in the proportion of cells in G1, and depletion of both p21 and SCML2 had an additive effect (Figure 4C). If the effect of SCML2 in G1/S progression were solely mediated through the function of p21 on CDK2/CYCE, a similar effect would be expected between the single and double knockdowns. However, the siRNA against p21 results in a stronger depletion of p21 than the reduction of SCML2 alone (Figure 4E), and this may be affecting other functions of p21, such as inhibition of PCNA activity [43],[44]. In the absence of p21, other members of the Kip family of inhibitors, such as p27, can compensate for the inhibition of the CDK/CYC complexes (see below), but not for these additional functions. We noted that knockdown of SCML2 in the absence of p21 induces a greater reduction in the levels of p27 than knockdown of SCML2 alone (Figure 4E), and this could result in an additive acceleration of passage through G1 through the combined regulation of CDK2/CYCE activity and other p21-dependent processes such as PCNA activity. The double knockdown of p21 and SCML2 did not allow us to firmly conclude if SCML2 functions in G1/S progression, and the effect of SCML2 depletion in the cell cycle is reminiscent of the overexpression of CYCE. Thus, we decided to analyze the effect of a simultaneous reduction of SCML2 and CYCE2, the CYCE homologue detected in our initial purification (Figure 1D and E). Depletion of CYCE2 alone did not have a significant effect on the cell-cycle distribution in U2OS cells, but it partially rescued the effect of SCML2 knockdown (Figure 4D). The double knockdown of CYCE2 and SCML2 did not change the levels of p21 or p27 when compared to the knockdown of SCML2 alone (Figure 4E).

The changes in the cell-cycle profile upon SCML2 knockdown (Figure 4B) are similar to the effects of CYCE overexpression. Together with the decreased p21 protein levels (Figure 4A) and the effect of the double knockdown of SCML2 and CYCE2 (Figure 4D), these data suggest a role for SCML2 in delaying the progression through G1 via regulation of CDK2/CYCE activity. To confirm this point, we knocked down SCML2 in U2OS cells, arrested them in mitosis, and then monitored their progression through G1 after release (Figure S8A). In the case of control siRNA, the cells progressed from mitosis to G1 in ∼4 h and began S phase in 12–14 h (Figure S8A). Although SCML2 knockdown did not affect the exit from mitosis (Figure S8A), by 16 h a larger proportion of cells were in S phase compared to the control (Figure 4F). In fact, entrance into S phase occurred significantly faster (2 to 4 h earlier than control cells) when the levels of SCML2 were reduced, particularly in the case of siRNA #2 (Figure 4G). Similar results were obtained by monitoring the entry into S phase using EdU staining (Figure S8B). Again, the magnitude of the acceleration of G1 passage is similar to what has been previously reported upon overexpression of CYCE1 [45].

### SCML2B Regulates p21 Accumulation and CYCE2 Activation During G1

**Figure 5:**
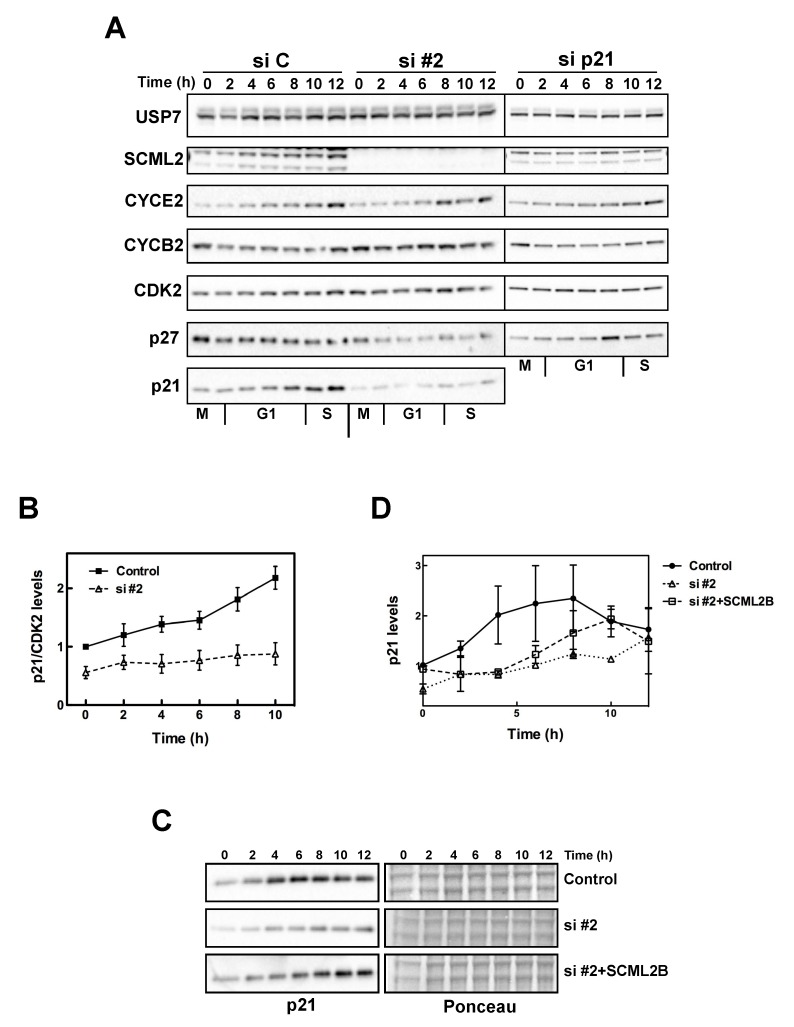
Regulation of G1/S progression by SCML2. (A) Western blot analysis of nuclear extracts isolated from U2OS cells transfected with siRNA against either SCML2 (#2) or p21 (si p21) or a control siRNA (si C). Cells were collected every 2 h, as indicated, after release from a mitotic arrest induced with nocodazole. The extracts were analyzed using antibodies specific to USP7 (loading control), SCML2, CYCE2, CYCB2, CDK2, p27, and p21. (B) Densitometric analysis of the levels of p21 at different points after release from mitosis in cells treated with a control siRNA or specific for SCML2 (si #2). The expression was normalized to the levels of CDK2, and the mean of four different experiments is shown. (C) Western blot analysis of nuclear extracts isolated from U2OS cells transfected with a control siRNA and plasmid (Control), with an siRNA against SCML2 and a control plasmid (si #2) or an siRNA against SCML2 and a plasmid expressing SCML2B resistant to the siRNA (si #2+SCML2B). Cells were collected every 2 h, as indicated, after release from a mitotic arrest induced with nocodazole. The extracts were analyzed using antibodies specific to p21. Ponceau staining is shown as loading control. (D) Densitometric analysis of the levels of p21 at different time points after release from mitosis in two independent experiments.

We further analyzed the function of SCML2B during the progression from G1 to S phase. Thus, we arrested U2OS cells in mitosis with nocodazole and analyzed if depletion of SCML2 or p21 affected the levels of cyclins and their inhibitors during G1 progression. In control-treated cells, the levels of SCML2 increased during G1 (Figure 5A), confirming that its expression is highest in S phase (Figure S6B). Interestingly, the increase in SCML2 levels paralleled the accumulation of p21, and was not detected in the absence of p21 (Figure 5A). Correspondingly, the accumulation of p21 during G1 was blocked when SCML2 was knocked down (Figure 5A–B). Additionally, CYCE2 levels increased prematurely, and CYCB2 reduction after mitosis exit was impaired in the absence of SCML2 (Figure 5A). In control-treated cells and SCML2-depleted cells, the levels of p27 remained constant or slightly decreased during G1 progression. In contrast, the levels of p27 increased in G1 when p21 was depleted, suggesting that it may compensate for its loss (Figure 5A). The changes in the levels and accumulation of p27 could further explain the accumulative decrease in the percentage of cells in G1 observed upon double knockdown of SCML2 and p21 (Figure 4C).

These data suggest that SCML2 modulates the accumulation of p21 and CDK2/CYCE2 complexes in G1. Accordingly, overexpression of SCML2B induced a slight increase in p21 in control cells growing asynchronously, and SCML2B also rescued the decrease in p21 upon SCML2 knockdown (Figure S9A). Overexpression of SCML2A (to higher levels than those attained with SCML2B) had no effect on the levels of p21 in control cells, and only partially restored the levels of p21 in the absence of endogenous SCML2 (Figure S9A), suggesting that it can potentially contribute to p21 regulation, at least in an overexpression setting. Further, upon exit from mitosis, overexpression of SCML2B was sufficient to restore the accumulation of p21 during G1 in the absence of the PRC1-associated SCML2A isoform (Figure 5C). The effect is not complete, as the kinetics of p21 accumulation are delayed when SCML2B is overexpressed compared to control cells (Figure 5D), suggesting that additional indirect effects may be contributing to the regulation of G1 progression by SCML2.

**Figure 6:**
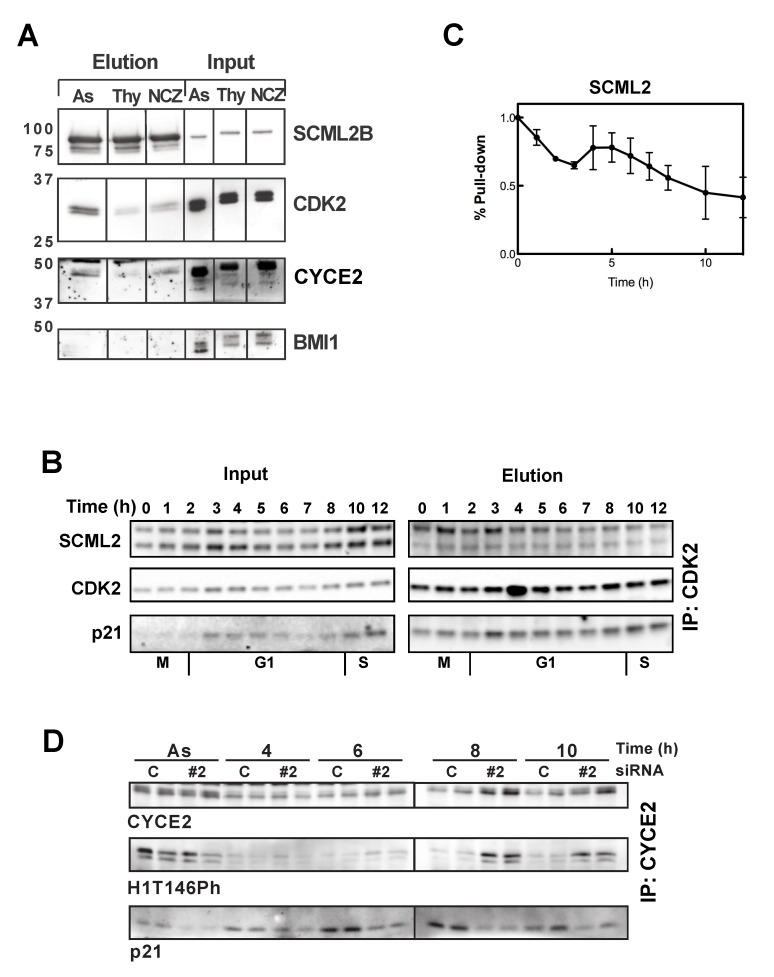
SCML2 interacts with CDK2/CYCE2 complexes in G1 and regulates their activation. (A) Western blot analysis of the SCML2B pull-down in 293 cells expressing FS-SCML2B, from asynchronously growing cells (As), and cells treated with thymidine (Thy) or nocodazole (NCZ). The pull-down was analyzed using SCML2, CDK2, CYCE2, and BMI1 antibodies. Shown on the right is 1.5% of the input. (B) Immunoprecipitation of CDK2 in cells treated with a control siRNA (C) or with siRNA specific for SCML2 (si #2) or p21 (p21), arrested in mitosis with nocodazole and released for different times, as indicated. Two percent of the input is shown (left) along with the elution (right). (C) Densitometric analysis of the amount of SCML2 pulled down by CDK2 at different time points after exit from mitosis, as in (B); the percentage of pull-down was normalized versus the input material and related to the amount of CDK2 in the pull-down. (D) Quantification of the kinase activity of CYCE2-associated complexes pulled down from control-treated U2OS cells and cells treated with siRNA #2 for SCML2, either growing asynchronously (As) or at different times after release from arrest in mitosis with nocodazole, as indicated. The reaction was performed in replicates, and the experiment was repeated twice. The levels of H1T146Ph were measured by Western blot with specific antibodies, and the levels of CYCE2 and p21 in the reaction are also shown.

Although our original purification of the SCML2B complex did not recover p21, the experiments above suggest a functional link between the two. To verify that p21 associates with SCML2B *in vivo*, we first fractionated nuclear extracts from HeLa cells and analyzed co-elution by size exclusion chromatography. The resulting profiles demonstrated that both p21 and p27 associated with SCML2B, CYCE2, and a fraction of CDK2 (Figure S9B). CDKs and cyclins were also detected in other fractions, but p21 and p27 peaked in the same high molecular weight fraction as SCML2B, which, together with our *in vitro* results (Figure 2 and Figures S2 and S3), suggested that SCML2 binds to p21/p27 and CDK/CYC complexes co-operatively. In this sense, the pull-down of p21 in nuclear extracts only recovers a very small amount of SCML2 (Figure S1F), indicating that in cells SCML2 interacts mainly with CDK2/CYCE. Even if the interaction of SCML2 with p21 is not direct, we reasoned that p21 could be modulating the binding of CYC/CYC and changing through the cell cycle, similar to the effect observed *in vitro*. Indeed, CDK2 co-precipitated with FS-SCML2B in asynchronously growing 293 cells (most of which are in G1). This interaction was less pronounced in cells arrested in S phase with thymidine or in mitosis with nocodazole (Figure 6A), suggesting that the association of SCML2B with CDK2 occurred mainly in G1. A detailed analysis of the interaction in extracts from U2OS cells upon release from mitosis revealed that the binding of SCML2 to CDK2 is high at the exit of mitosis (30 min after release from the mitotic arrest) (Figure 6B–C). The percentage of SCML2 pulled down by CDK2 decreases during early G1, and then peaks again coinciding with the onset of p21 accumulation and association of p21 to CDK2 (Figure 6B–C). During the G1/S transition the binding of SCML2 to CDK2 is progressively reduced (Figure 6B–C), in parallel with the onset of p21 accumulation (Figure 5A).

Next, we analyzed this interaction in cells depleted for SCML2 or p21, quantifying the amount of these proteins pulled down by CDK2, and normalizing by the input and the levels of CDK2 in the elution. The percentage of SCML2 pulled down in cells depleted for p21 was calculated compared to control-treated cells, showing that the depletion of p21 decreases its association to CDK2 (Figure S10A, upper panel). The pull-down of p21 is also reduced when SCML2 is depleted (Figure S10A, lower panel). While the association of p21 increases around 4 h in control-treated cells, this effect is only seen at later time points (10–12 h) in the absence of SCML2 (Figure S10A, lower panel), confirming that the association of p21 with CDK2 is delayed in the absence of SCML2. As a whole, these results are in line with the *in vitro* experiments (Figure S3) showing that low amounts of p21 stimulate the interaction of SCML2B with CDK2/CYCE complexes. At 4 h after exit from mitosis the presence of low amounts of p21 and SCML2 may co-operate in the binding to CDK2/CYCE complexes. Because of this initial interaction, p21 accumulates and, later in G1, inhibits CDK2, independent of SCML2. Our results also suggest that there are additional functions for SCML2 in the exit from mitosis.

The results presented above indicated that in the absence of SCML2, the interaction of p21 with the accumulating CDK2/CYCE is not established in a timely manner, resulting in a premature activation of these complexes. To address this possibility, we measured the kinase activity of CYCE2-associated complexes in cells treated with siRNA against SCML2 (si#2), growing asynchronously or released from mitotic arrest. Using H1e as a substrate, we could not detect a substantial difference in the activity of CYCE2 in asynchronously growing U2OS cells in the absence or presence of SCML2 (Figure 6D, As). In contrast, in cells arrested in mitosis and released for different times, we detected a faster and stronger activation of CYCE2-asssociated kinases (Figure 6D, 8–10 h), in agreement with the data showing faster entry into S phase upon SCML2 knockdown, as measured by PI and EdU staining (Figure 4F and Figure S8B, respectively). Interestingly, in control cells an increase in the association of CYCE2 and p21 was observed around 6–8 h after release, prior to the increase in CDK2/CYCE2 activity and before the maximal levels of p21 are achieved (Figure 6D). Again, the data suggest that the presence of SCML2 potentiates the initial interaction of p21 with CDK2/CYCE2 to establish an effective inhibition of the complex. In contrast, in cells with reduced levels of SCML2, the association of CYCE2 with p21 did not change during G1 as the accumulation of p21 is impaired. As a consequence, the inhibition of CDK2/CYCE2 complexes is also blocked (Figure 6D).

**Figure 8:**
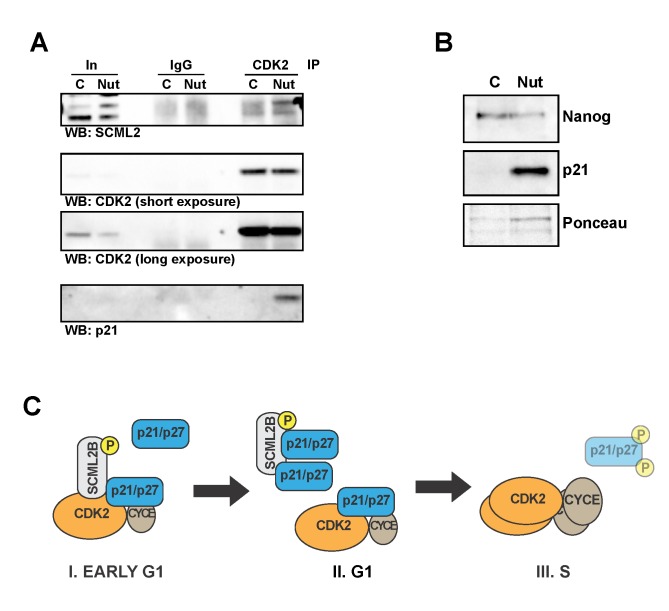
ES cell differentiation modulates the interaction of SCML2 with CDK2. (A) Immunoprecipitation of CDK2 in nuclear extracts from control-treated H9 cells (C) or cells treated with 15 µM Nutlin for 3 d (Nut). The material pulled down was analyzed with specific antibodies for SCML2, CDK2, and p21. Immunoprecipitation with a nonspecific IgG is used as control, and 1% input is shown (In). (B) Western blot analysis of the levels of Nanog and p21 in nuclear extracts of control H9 cells (C) and cells treated with 15 µM Nutlin for 3 d (Nut). Ponceau staining is shown as loading control. (C) Model for the proposed mechanism of action of SCML2B on the regulation of G1 progression. SCML2B and p21 or p27 cooperatively bind and inhibit CDK2/CYCE complexes in early G1 (step I, left), preventing their premature activation. As p21 and p27 levels increase, their association with CDK2/CYCE becomes more stable and independent of SCML2B, and restricts progression into S phase (step II, middle). Over time, increasing amounts of CDK2/CYCE lead to the phosphorylation of p21 and p27 and promote their degradation to allow the entry into S phase (step III, middle). Our data show that in the absence of SCML2B step I is missing, which results in a less effective step II and an accelerated progression to step III.

### SCML2B Stabilizes p21 in Early G1

**Figure 7:**
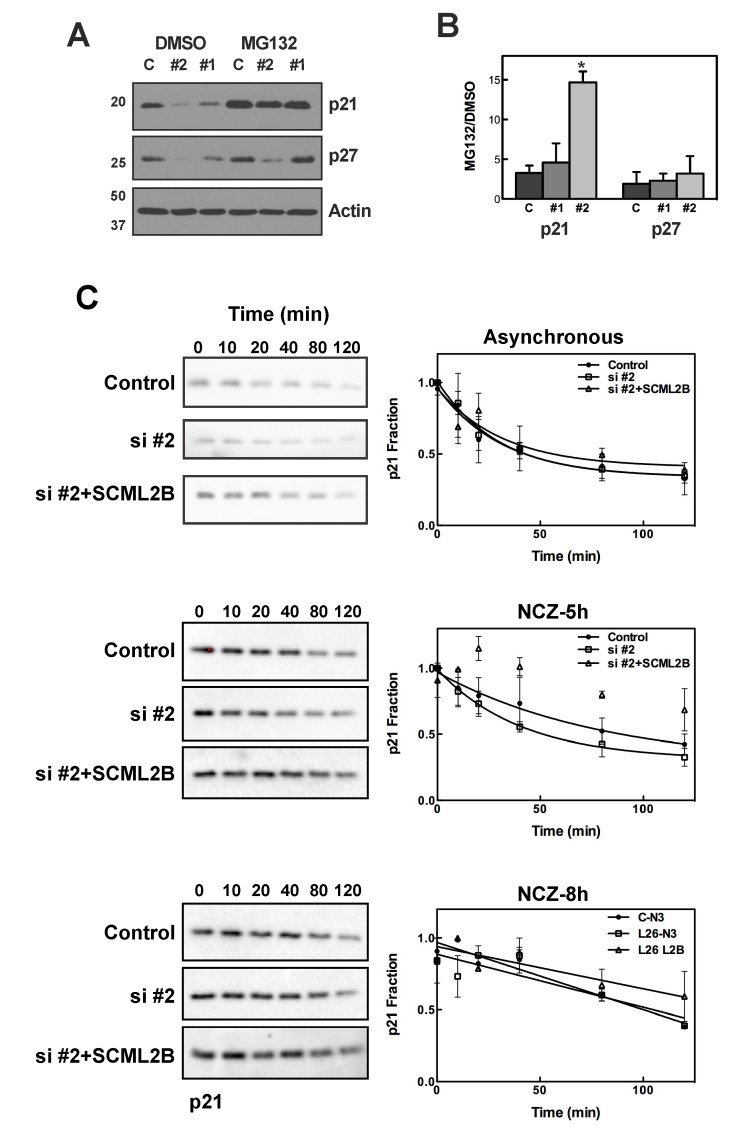
SCML2B stabilizes p21. (A) Western blot analysis of p21, p27, and actin, using whole cell extracts from U2OS cells treated with two different siRNAs (#1, #2) for SCML2 or a control siRNA (C) for 72 h, and then incubated in the presence of 5 µM MG132 or DMSO. (B) Quantification of the densitometric analysis for p21 and p27, from three different experiments performed as in (A). The intensity was corrected for the expression of actin, and the ratio of the levels after MG132 treatment relative to those of DMSO treatment is shown for each siRNA. *p<0.01 (C) U2OS cells were transfected with a control siRNA and plasmid (Control), with an siRNA against SCML2 and a control plasmid (si#2) or an siRNA against SCML2 and a plasmid expressing SCML2B resistant to the siRNA (si#2+SCML2B) for 48 h. Cells growing asynchronously (top panel) or released from an arrest in mitosis (5 h, middle panel, and 8 h, bottom panel) were then incubated with 25 µg/ml cycloheximide, and whole cells extracts were analyzed at different time points by Western blot with antibodies specific for p21. Densitometric quantification of the levels of p21 from two different experiments, each of them loaded in duplicate, is shown. The data were fitted to a one phase decay equation.

Our results suggest that SCML2B contributes to the stabilization of p21 upon exit from mitosis and to an efficient inhibition of CDK/CYCE. Consistent with this possibility, treatment with the proteasome inhibitor MG132 reversed the reduction in p21 protein levels caused by SCML2 knockdown, while only a limited effect was detected for p27 (Figure 7A–B). SCML2 knockdown had no effect on the levels of unrelated proteins, USP7 and PR-Set7 (Figure S10B). Proteasome inhibition did not affect USP7 protein levels, although it did result in increased expression of PR-Set7, as previously reported [46] (Figure S10B). The half-life of p21 decreased from ∼22 min in control cells to ∼20 min after knockdown of SCML2, and this effect was rescued by an siRNA-resistant version of SCML2B (Figure 7C, top). Given that SCML2 specifically interacts with CDK2 during a narrow temporal window in G1 progression, we speculate that a stronger effect could be detected at this time. As expected, the half-life of p21 was greatly increased in synchronized cells 8 h after release from mitosis (half-life over 60 min), independent of the presence or absence of SCML2 (Figure 7C, bottom). However, at 5 h after mitosis knockdown of SCML2 resulted in much less stable p21 (half-life of ∼28 min versus ∼56 min in siRNA control-treated cells; Figure 7C, middle). Importantly, overexpression of SCML2B alone completely rescued this phenotype (Figure 7C, middle), reinforcing the notion that the effect of SCML2 proteins on p21 stability is independent of PRC1. Taking into account the time frame for CDK2/CYCE2 activation, our results suggest that the doubling of the half-life of p21 is important to establish an inactive complex around 6 h after release, and to avoid premature activation (Figure 6D). Thus, SCML2B is required for stabilization of p21 in early G1, when p21 levels are limiting, and stimulates the formation of an inactive complex.

### The Interaction of SCML2 with CDK2 Is Regulated During Differentiation

PcG proteins play an essential role in the modulation of self-renewal and differentiation of embryonic stem (ES) cells. p21 has been proposed to mediate the induction of differentiation upon treatment with Nutlin, an inhibitor of MDM2 that increases the levels of p53 [47]. In contrast, other differentiating agents such as retinoic acid do not induce the expression of p21 [47]. We analyzed whether the induction of differentiation affected the interaction of SCML2 with CDK2 and if this effect was dependent on the induction of p21. Pull-down of CDK2 in control H9 ES cells shows a weak interaction between SCML2 and CDK2 (Figure 8A and Figure S10C). Treatment of H9 cells with 15 µM Nutlin for 3 d increased the amount of SCML2 pulled down by CDK2 (Figure 8A), along with the induction of p21 and differentiation of the cells as assessed by reduced levels of Nanog (Figure 8B). In contrast to p21, the levels of p27 remained undetectable under these conditions. Treatment of cells with 30 µM retinoic acid for 3 or 5 d reduced the levels of p21 and increased the levels of p27 (Figure S10D), as previously reported [47], while inducing a strong decrease in the levels of Nanog (Figure S10D), confirming the differentiation of the cells. The treatment with retinoic acid slightly increased the interaction between SCML2 and CDK2 (Figure S10C), although the effect is smaller than the one observed with Nutlin treatment (Figure 8A). These results suggest that SCML2 may have an effect in the regulation of the cell cycle during the differentiation of ES cells, and this effect is likely modulated by the induction of p21, independent of differentiation itself.

In conclusion, our findings show that SCML2B associates with CDK/CYC/p21-p27 complexes and promotes the p21-mediated inhibition of CDK/CYC *in vitro*. *In vivo*, SCML2B contributes to the stabilization of p21 in early G1, and fosters both the accumulation of p21 and the establishment of an inactive CDK2/CYCE complex with p21 and SCML2B, even when limiting amounts of p21 are present. As a consequence, SCML2B inhibits the premature activation of CDK2/CYCE complexes, prolongs the duration of G1, and contributes to proper cell-cycle progression (Figure 8C). Our data further suggest a role for SCML2 on the regulation of the cell cycle of ES cells during differentiation, in coordination with p21.

## Discussion

PcG proteins ensure the epigenetic repression of lineage-specific genes that is necessary for the correct development of complex organisms [2]. Consequently, they are indispensable for the maintenance of pluripotency [48] and for the proper onset of differentiation programs [2]. The transition from pluripotent to specialized cells requires that the differentiation programs and the proliferation capacity of these cells be coordinately regulated [49]: as cells become specialized, their proliferation potential is progressively reduced, until they stop dividing when terminal differentiation is reached. In some cases, adult stem cells with a pluripotent state present a slow proliferation rate, allowing the generation of a pool of stem cells that can be expanded when needed. Therefore, it is not surprising that PcG proteins are involved in the transcriptional regulation of gene networks controlling the cell cycle, exerting a pivotal role in the interplay between differentiation and proliferation [50]. For example, in *Drosophila*, the *cyclin A* gene is repressed by PcG proteins through a Polycomb Response Element within its promoter, linking the stable repression of *cyclin A* with the differentiation process [51]. In mammals, both PRC1 and PRC2 bind to and repress the *INK4a/Arf* locus [52]–[54], which encodes several proteins involved in cell-cycle regulation. Recently it has been shown that CBX7, a component of PRC1, represses the *CYCE1* gene [55].

Most of the evidence for cell-cycle regulation by PcG proteins arose from studies of indirect effects through transcriptional repression of specific genes. Only very recently, PSC, a component of PRC1, has been reported to affect the stability of CYCB in *Drosophila* [32]. Interestingly, this activity of PSC does not depend on the PRC1 complex. In mammals, PRC1 has been proposed to regulate the stability of MDM2 and/or p53 [30],[31], which can indirectly modulate the cell-cycle machinery. To our knowledge, the binding and regulation of CDK/CYC complexes by SCML2B represent the first direct biochemical link between the *Polycomb* axis and cell-cycle progression in mammals. The presence of two isoforms potentially allows SCML2 to coordinate PRC1 function with cell-cycle regulation: SCML2A associates with PRC1 through its SPM domain and regulates its recruitment to chromatin (Bonasio et al., submitted), while SCML2B is present in the soluble nuclear fraction where it modulates the activation of CDK2/CYCE complexes. The concerted regulation of the expression of both isoforms may establish a dual activity on transcription and the cell cycle, similar to the dual function of PSC in *Drosophila* [32]. A role for PRC1 in the DNA damage response is also becoming prominent [27], supporting the function of PcG as an essential axis to control cell fitness through cell division and differentiation.

Consistent with our *in vitro* and biochemical observations, we show that SCML2B functions *in vivo* during the G1/S checkpoint, slowing the progression of cells through G1. SCML2B interacts with CDK2/CYCE complexes at early G1, when p21 is in limiting amounts. Increased levels of p21 ensure CDK/CYC inhibition and most likely impair the interaction of SCML2B with CDK/CYC complexes by blocking the binding surfaces on SCML2B, which would explain why excess amounts of p21 do not result in increased SCML2B binding (Figure S3). Additionally, most of the CDK2/CYCE complexes are bound by p21 or p27 in cells [56],[57] (Figure 6 and Figure S10), and the small pool of free CDK2/CYCE is critical for progression from G1 to S phase. This is in good agreement with the recently revisited model for cell-cycle progression. In this model, sequential increases of CDK activity determine the progression into S phase or mitosis [58]. At early G1, CDK2/CYCE complexes should be tightly controlled to avoid CDK activity from rising above the threshold required for progression into S phase, and therefore the binding of SCML2B to a small fraction of total CYCE could be relevant for G1/S progression [56],[57]. In this context, we propose that SCML2B promotes the interaction of p21 with the small pool of free CDK2/CYCE. Our data show that SCML2 mainly interacts with CDK2/CYCE *in vivo*, and this binding in early G1 promotes p21 stabilization, preventing the premature activation of the complex and controlling progression through G1, which is accelerated upon SCML2 depletion (Figure 6). Reciprocally, the presence of p21 is necessary for the function of SCML2, indicating that the cooperative binding of CDK2/CYCE, p21, and SCML2B observed *in vitro* is also relevant in cells. This is also consistent with the increased interaction of SCML2 with CDK2 in differentiated versus control ES cells. The cell cycle in ES cells presents a very short G1 phase, with no checkpoints controlling G1/S transition. This is due to high and constitutive activation of CDKs, with undetectable levels of Ink4a and Kip CDK inhibitors [59]. Overexpression of p21 can arrest the cell cycle of human ES cells inducing their differentiation [60], and elevated p21 levels drive the differentiation of cells upon treatment with Nutlin, correlating with the increased interaction between SCML2 and CDK2. Our results suggest a role for SCML2 in the coordination of differentiation of ES cells and the modulation of the cell cycle, a system where the restoration of the G1 checkpoint is essential to allow cells to differentiate.

Additionally, we show that SCML2 is itself a target of the kinase activity of CDK/CYC and that its phosphorylation occurs preferentially during mitosis. Although our results show that phosphorylation by itself is not enough to affect the interaction of SCML2B with p21, the presence of Pin1 leads to a decreased *in vitro* interaction between these proteins. In this way, a regulatory feedback loop can be established to restrict the functions of SCML2 to early G1. Other PcG proteins are also regulated by phosphorylation by the CDKs, as is the case of EZH2 (a PcG protein that is part of the PRC2 complex) [61]–[64]. Taking into account that the binding of is maximal upon exit from mitosis, we cannot rule out that SCML2B plays additional roles in other phases of the cell cycle, such as mitosis. A detailed analysis of the phosphorylation of SCML2 in cells will be required to elucidate the interplay between the phosphorylation and the association to CDK2-containing complexes.

Proteins that regulate cell-cycle progression are often targets of mutations and epimutations during cancer development and progression. Indeed PcG proteins often act as tumor suppressors. For example, removal of PRC1 components from *Drosophila* eye imaginal discs leads to increased proliferation and tumor-like phenotypes, possibly via deregulation of the Notch or JAK/STAT signaling pathways [65],[66]. In mammals, MEL18 and CBX7 have also been proposed to act as a tumor suppressor in prostate cancer [55],[67]. The effects of SCML2B depletion on the cell cycle reported here suggest that SCML2B may also function as a tumor suppressor. In its absence, cells enter S phase too early, leading to faster proliferation, and potentially increasing genome instability. Although a possible role for the *SCML2* gene in cancer has already been suggested [26],[68], the lack of functional information on the proteins that it encodes has hampered the elucidation of its mechanism of action. Single base and frame-shift mutations have been reported in different kinds of cancers in several databases (http://dcc.icgc.org/web/), but these alterations have remained unexplored. Indeed, the absence of *scm* in *Drosophila* induces an increase in proliferation similar to that associated with defects in PRC1 components [10],[66], and human *SCML2* and other MBT-containing genes are focally deleted in medulloblastomas [26] and breast cancer [69]. Deregulation of the expression of *SCML2* has also been observed in acute myeloid leukemia and in several T-cell malignancies [68].

In light of these studies and our new observations, we propose that *SCML2* contributes to the regulation of the cell cycle. Based on our results we propose that *SCML2* exerts this regulatory function through its nucleoplasmic isoform, SCML2B, which stabilizes p21 and reinforces its inhibitory activity on CDK2/CYCE, avoiding a premature activation of the complex and thereby delaying the entry of cells in S phase.

## Materials and Methods

### Cell Lines, Extract Preparation, Transfections, and Antibodies

HeLa, U2OS, K562, and 293T-REx cells were grown in DMEM with 10% FBS, penicillin (100 IU/ml), streptomycin (100 µg/ml), and glutamine (300 µg/ml). Human H9 (WA09) embryonic stem cell lines were obtained from WiCell Research Institute and grown in TeSR medium (Stem Cell Technologies) with GELTREX (Life Technologies). For differentiation experiments, H9 cells were individualized by treatment with accutase (Millipore) and then treated with 15 µM Nutlin or 30 µM retinoic acid (Sigma) in DMEM/F12 medium (Invitrogen) supplemented with 20% Knockout Serum Replacement (Invitrogen), 1 mM L-glutamine, 0.1 mM nonessential amino acids, 55 µM β-mercaptoethanol, and 2.5 ng/ml bFGF (R&D). Cytosolic and nuclear extracts were prepared as previously described [70]. The nuclear pellet was extracted by solubilization in 50 mM Tris, pH 7.5, 8 M Urea, and 1% Chaps. Transfection of the siRNA for human SCML2 (#1 5′CCAAACGATCTCCTCAGCAAA, #2 5′CAGTATGTATTGCTACGGTTA, #3 5′GTTATATAGCTGTGTACCTGA, #4 5′CAGGAGATATTTATACTACGA) or for human p21 and CYCE2 (SMARTPool L-003471-00-0005 and L-003214-00-0005, from Dharmacon) was performed using lipofectamine RNAimax (Invitrogen) according to the manufacturer's instructions. Cotransfection of the different siRNA together with the empty pINTO vector or the pINTO-FSH-SCML2A, pINTO-FSH-SCML2B was performed using Lipofectamine 2000 (Invitrogen) according to the manufacturer's instructions. 293T-REx cells were transfected with the pINTO-FS-SCML2B [71] plasmid using PEI, and clones were selected in the presence of 5 µg/ml blasticidin (InvivoGen) and 100 µg/ml Zeocin (Invitrogen). SCML2B expression was induced with 1 µg/ml doxycycline for 24 h. For proteasome inhibition, cells were treated with 5 µM MG132 (or DMSO in the control cells) for 4 h. For the determination of the half-life of p21, cells were incubated with 25 µg/ml cycloheximide (Sigma) for 10/20/40/80/120 min.

Rabbit antibody against SCML2 was generated using a GST fusion protein of a central region of SCML2, and affinity purified. The antibodies against CDK2 (Santa Cruz, sc-163), CYCE2 (Epitomics, 1775-1), CYCB2 (Santa Cruz, sc-22776), p21 (Calbiochem), p27 (BD, 610242), histone H1T146-Phospho (Abcam, ab3596), USP7 (Bethyl, A300-033A), RNA polymerase II and PRSET7 (custom made), and CYCE and CYCB (kindly provided by Dr. Michele Pagano) were used for Western blot analysis and immunoprecipitation.

### SCML2B Purification

The purification of SCML2B is schematically depicted in Figure S1D. Briefly, nuclear extracts obtained from HeLa S3 cells were loaded onto a p11 column, and the bound material was eluted with increasing salt concentrations. SCML2 elutes mainly at 0.3 M KCl. After dialysis, this fraction was then loaded onto a cation exchange DE52 resin. SCML2 remains in the flow-through, which was loaded onto an anion exchange CM-sepharose column. Bound material was eluted with 1 M NaCl, dialyzed, and loaded onto another anion exchange column, SP-sepharose. Step elution with increasing NaCl concentration recovered SCML2 in the 0.3 M fraction. This material was dialyzed and subjected to a strong cation exchange chromatography using a MonoQ column. SCML2 was recovered in the flow-through and then loaded onto a Heparin affinity column. The bound material was eluted using a NaCl gradient (0.05 to 0.6 M). The fractions containing SCML2 were pooled, dialyzed, and then subjected to a strong anion exchange chromatography using a MonoS column. The bound material was eluted with a NaCl gradient (0.05 to 0.6 M). At this step the two isoforms of SCML2 separated in two different pools. The pool containing SCML2B was dialyzed and loaded onto a HiTrap SP-sepharose anion exchange column. A step elution with 1 M NaCl was performed to concentrate the sample, which was then fractionated on a Superdex200 size exclusion column. The fractions containing SCML2B (150–200 kDa) were dialyzed and then fractionated by affinity chromatography using a Heparin-5PW column. After elution with a NaCl gradient (0.04 to 0.6 M), SCML2B-containing material was dialyzed and then loaded onto a Hydroxyapatite column. Bound material was eluted with a phosphate gradient (0.01 to 0.5 M). The peak fraction for SCML2B was analyzed using SDS-PAGE and silver staining, and mass spectrometry was performed both in solution and from gel-excised bands. Further fractionation was performed using a Superdex200 size exclusion column, and protein elution was followed by Western blot analysis.

### Size Exclusion Chromatography

Proteins were incubated for 10 min at 30°C and fractionated on a Superdex 200 column (GE Healthcare) in 50 mM Tris, pH 7.5, 200 mM NaCl, and 10% glycerol. Nuclear extract from HeLa cells was fractionated on a Superose 6 column (GE Healthcare) in 50 mM Tris, pH 7.5, 200 mM NaCl, and 10% glycerol.

### *In Vitro* Kinase and Dephosphorylation Assays

Histone H1e from calf thymus (14–155, Millipore) was incubated with the indicated CDK/CYC complexes at 30°C in 50 mM Tris, pH 7.5, 10 mM MgCl_2_, 1 mM DTT, 5 mM β-glycerophosphate, 1 mM sodium orthovanadate, and 1 mM ATP. The reaction was stopped after 30′ for end-point assays or after 2′ for the kinetics assay by addition of sample buffer. Dephosphorylation assays were carried out for 2 h at 37°C with Antarctic phosphatase (New England Biolabs).

Recombinant His-SCML2B was incubated *in vitro* in the presence of CDK2/CYCE, CDK1/CYCB, Aurora kinase A, or without kinase at 30°C in 50 mM Tris, pH 7.5, 10 mM MgCl_2_, 1 mM DTT, 5 mM β-glycerophosphate, 1 mM sodium orthovanadate, 1 mM ATP, and 1 µCi of γ^32^P-ATP (Perkin-Elmer, 3000 Ci/mmol).

The activity of CYCE2 in nuclear extracts was measured using complexes immunoprecipitated with an anti-CYCE2 antibody (Epitomics). Briefly, the antibody was coupled to Dynabeads (Invitrogen) in the presence of 1 mg/ml BSA. Nuclear extracts (100 µg) were incubated with the beads in 50 mM Tris, pH 7.5, 175 mM NaCl for 1 h at 4°C. The beads were washed 3 times with 50 mM Tris, pH 7.9, 200 mM NaCl, and 0.05% Igepal CA630 (Sigma-Aldrich); once with 50 mM Tris, pH 7.9, 100 mM NaCl; and then resuspended in 50 µl of the same buffer. We incubated 5 µl of the immunoprecipitated material with 150 ng of histone H1e, and the reaction was carried out as described above.

### Cell-Cycle Synchronization and Analysis

For double thymidine block, cells were incubated for 16 h with 2.5 mM thymidine, released into thymidine free medium for 9 h, and incubated again for 16 h with 2.5 mM thymidine. Arrest in mitosis was performed with 0.04 µg/ml nocodazole for 16 h, and then cells were released for 10 min. Arrest in S phase was performed with 2.5 mM thymidine for 16 h. For serum starvation, cells were incubated for 24 h in the absence of serum. Cells were trypsinized, fixed in 60% ethanol, and incubated with 0.09 mg/ml RNase A and 35 µg/ml propidium iodide overnight at room temperature. PI staining was detected in a FACscalibur (BD) flow cytometer, and the data were analyzed with FlowJo software. Progression into S phase was measured by incubating U2OS cells with EdU for 5 min at 37°C. EdU incorporation was measured using the Click-iT kit (Invitrogen), following the manufacturer's instructions.

### SCML2B Pull-Down from 293T-REx Cells

After induction of SCML2B with 1 µg/ml doxycycline for 24 h, cells were incubated in complete medium, arrested in the presence of 1 µg/ml doxycycline with either 2.5 mM thymidine or 0.04 µg/ml nocodazole for 16 h, or treated in the presence of 1 µg/ml doxycycline together with different concentrations of roscovitine for 8 h. Nocodazole-treated cells were then released for 10 min in complete medium. The cells were collected and nuclear extracts were obtained as described above. Nuclear extract was diluted to 1 mg/ml in 50 mM Tris, pH 7.9, 200 mM NaCl, and incubated with Strep-Tactin (IBA) beads for 1 h at 4°C. The beads were washed with 50 mM Tris, pH 7.9, 500 mM NaCl, and 0.1% Igepal CA630 (Sigma-Aldrich), and then with 50 mM Tris, pH 7.9, and 200 mM NaCl. SCML2B and associated proteins were eluted with 2 mM Biotin in 50 mM Tris, pH 7.9, and 200 mM NaCl. For mass spectrometry analysis, SCML2B was separated by SDS-PAGE, the gel was silver stained (SilverQuest, Invitrogen), and the band was excised.

### Mass Spectrometry Analysis of SCML2B Phosphorylation

Total SCML2B from *in vitro* phosphorylation reactions and excised SCML2B bands from Strep pull-downs were digested with trypsin. Phospho-peptides were identified and the position of the phosphorylation was determined by MSMS. For quantification of the phosphorylation in each peptide, the samples were divided in two, one was mock treated, and the other was dephosphorylated with lambda phosphatase (New England Biolabs), following the manufacturer's instructions. To control for loading and experimental variability, an internal standard of unphosphorylated ^15^N-His-SCML2B tryptic digest was added to each sample prior to sample division and phosphatase treatment. The area of the peak corresponding to the unphosphorylated peptide was measured, and the percentage of phosphorylation was calculated as follows:
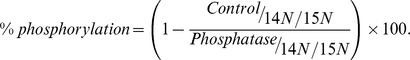



## Supporting Information

Figure S1 **Fractionation of SCML2A and SCML2B in 293-TRex cells and purification scheme for SCML2B starting with HeLa nuclear extract.** Related to Figure 1. (A–B) Stable clones of 293-TRex cells carrying an inducible plasmid for the expression of SCML2A (A) or SCML2B (B) were treated with different concentrations of doxycycline, as indicated, for 1 d. The presence of SCML2A and SCML2B in the cytosolic (Cyt), nuclear extract (NE), or nuclear pellet (NP) fractions was measured by Western blot. RNA polymerase II (O, hyperphosphorylated form; A, hypophosphorylated form) and tubulin are shown as controls for the fractionation. (C) Western blot analysis of nuclear extracts of HeLa and Jurkat cells using antibodies specific for SCML2A or recognizing both isoforms of SCML2. (D) Schematic representation of the chromatographic purification of SCML2B from HeLa nuclear extract. (E) Imperial Blue staining of the fractions from the hydroxyapatite column. The arrow indicates the peak for SCML2 elution and the numbers the regions that were cut for mass spectrometry analysis. (F) Immunoprecipitation of CDK2, SCML2 and p21 from nuclear extracts of HCT116 cells. Two percent of the input is shown along with the elution of each immunoprecipitation. A nonspecific IgG pull-down is shown as control. A short exposure of the Western blot detecting SCML2, CDK2 and p21 is shown on the left, and a log exposure on the right.
(TIFF)

Figure S2 **Purification of recombinant proteins and** ***in vitro*** **pull-down of SCML2B with CDK/CYC/p27 complexes.** Related to Figure 2. (A) Sf9 cells were co-infected with baculoviruses for the different CDK/CYC combinations (kindly provided by Dr. Robert Fisher). The cells were homogenized (H) and the complexes were purified through the cyclin His tag using a Ni-NTA column. The column was washed with 30 mM imidazole (W) and the complexes were eluted with 100 and 250 mM imidazole (Elution, E). The purified complexes were dialyzed against 50 mM Tris, pH 7.5, 50 mM NaCl, and 10% glycerol. (B) GST-p27 was expressed in BL21(DE3) cells and purified using a glutathione-sepharose column. An in-column digestion was performed with the GST-prescission protease, and the digested p27 was washed away from the column (Elution). Purified p27 was dialyzed as in (A). (C) His-SCML2B was expressed in BL21(DE3) cells and purified through a Ni-NTA chromatography. After washing with 30 mM imidazole, the protein was eluted with 250 mM imidazole. This fraction (E_0_) was further purified in a MonoS column and eluted with a salt gradient (Elution). The fractions containing the full-length His-SCML2B were pooled and dyalyzed against 50 mM Tris, pH 7.5, 100 mM NaCl, and 10% glycerol. (D–F) CDK/CYC complexes were incubated first with p27 and then with SCML2B. The proteins were pulled down using unspecific IgG and SCML2- or CDK2-specific antibodies. Five percent of the input is shown along with the flow-through (FT) and bound (E) fractions. The pull-downs were analyzed by Western blot using specific antibodies for SCML2, CDK2, or p27, as indicated. SCML2B interaction with CDK2/CYCE, p27, and CDK2/CYCE/p27 (D), CDK2/CYCA (E), and CDK1/CYCB/p27 (F).
(TIFF)

Figure S3 **Analysis of the binding of SCML2B with CDK2/CYCE/p21.** Related to Figure 2. (A) Schematic representation of SCML2A and SCML2B isoforms, together with the SCML2 fragments employed in the assays. (B) CDK2/CYCE complexes were incubated first with GST alone or GST-p21, and then with SCML2B. The proteins were pulled down using Glutathione sepharose beads. Five percent of the input is shown (a darker exposure is shown for the GST to visualize GST-p21, Input LE) along with the flow-through (FT) and bound (E) fractions. The pull-downs were analyzed by Western blot using specific antibodies for SCML2, GST, or CDK2, as indicated. (C) CDK2/CYCE complexes were incubated first with GST alone or GST-p21, and then with different fragments of SCML2B. The proteins were pulled down using Glutathione sepharose beads. Five percent of the input is shown along with the bound fractions. The pull-downs were analyzed by Western blot with antibodies for SCML2 (Ran-SPM fragment) or by silver staining (MBT-DUF and MBT fragments). (D) CDK2/CYCE complexes were first incubated with SCML2B, and then increasing amounts of GST-p21 (0.5, 1, 2, and 5× molar ratio with CDK2/CYCE) or GST alone (−) were added. Complexes were pulled down with antibodies against CDK2, and the bound material was analyzed by Western blot with antibodies specific for SCML2 or GST. Five percent of the input material (In) is shown. (E–F) CDK2/CYCE complexes were incubated with two different amounts of SCML2B, and then increasing GST-p21 or GST (−) was added. Complexes were pulled down with CDK2 antibody (E) or Sepharose-Glutathione beads (F), and the bound material was analyzed by Western blot with antibodies specific for GST or CDK2. Five percent of the input is shown (In).
(TIFF)

Figure S4 **Size exclusion chromatography of CDK2/CYCE and CDK2/CYCE/p27 complexes in the presence of SCML2B. Related to** **Figure 2** **.** CDK2/CYCE complexes were incubated with p27 or SCML2B to form CDK2/CYCE/p27 and CDK2/CYCE/SCML2B complexes. Further incubation of the former complex with SCML2B yielded CDK2/CYCE/p27/SCML2B. The complexes were fractionated on Superdex 200 size exclusion chromatography. Elution profiles for the proteins indicated after fractionation of either the CDK2/CYCE/SCML2B complex (left panel), or the CDK2/CYCE/p27/SCML2B complex (right panel), as analyzed by Western blot.
(TIFF)

Figure S5 ***In vitro*** **kinase assay and effect of SCML2B.** Related to Figure 2. (A) CDK/CYC complexes were incubated at 30°C for 60 min with 100 ng of histone H1e in the reaction buffer, in the absence or presence of ATP. The phosphorylation of histone H1e was followed using an antibody specific for T146Phospho. The treatment of recombinant histone H1e with Antarctic phosphatase 30°C for 60 min is shown as a control; the basal phosphorylation of the substrate is negligible. (B) The kinase assay was performed as in (A) in the presence of increasing p27. (C) Kinase assays were performed employing the indicated fractions from the S200 column in Figure 3B. The levels of phosphorylated H1e (H1T146Ph) were measured by Western blot and were normalized for the levels of CDK2 in the fractions. (D) Western blot analysis of the fractions assayed in (C), with antibodies specific for SCML2, CDK2, p27, and phosphorylated H1e. (E) Relative kinase activity of fractions containing SCML2B compared to fractions without SCML2B (C) for CDK2/CYCE and CDK2/CYCE/p27 complexes.
(TIFF)

Figure S6 **Expression and phosphorylation of SCML2 during the cell cycle.** Related to Figure 3. (A) HeLa cells were synchronized at the G1/S border with a double thymidine block. Progression through the cell cycle was measured by staining with propidium iodide and analysis by flow cytommetry. (B) Western blot analysis of nuclear extracts isolated from HeLa cells every 2 h, as indicated, after cells were released from a double thymidine block. Nuclear extract from asynchronous (AS) cells is also shown. The blot was probed with antibodies specific to SCML2, CYCE2, and p27, and the membrane was stained with Ponceau Red for loading control, as indicated. (C) Nuclear extracts from HeLa cells growing asynchronously (As) or arrested in G0 (SS), G1/S (Thy), or M (NCZ) were probed for SCML2 in Western blots (upper panel). A densitometric analysis of the protein species as a function of mobility is shown (lower panel). The black line indicates the position of the peak in nocodazole-treated cells, and the red line indicates the position of the peak in thymidine-treated cells.
(TIFF)

Figure S7 **Identification of residues of SCML2B phosphorylated by CDK2/CYCE or CDK1/CYCB** ***in vitro*** **. Related to** **Figure 3** **.** (A) Two examples of phospho-peptides within SCML2B after treatment with CDK2/CYCE or CDK1/CYCB, identified by mass spectrometry. No signal for these peptides was detected in control or Aurora kinase A–treated SCML2B (upper panel). The position of the phosphorylation was determined by MSMS (lower panel). (B) FS-SCML2B pull-down from 293 cells growing asynchronously (As) or treated with thymidine (Thy) or nocodazole (NCZ). The same elution in each condition is loaded several times, and the bands were excised, combined, and analyzed by mass spectrometry. (C) 293T-Rex cells expressing FS-SCML2B were treated with Roscovitine for 8 h at the indicated concentrations, and the cell cycle was measured by staining with propidium iodide and analysis by flow cytommetry.
(TIFF)

Figure S8 **Effect of SCML2 knockdown on progression into S phase.** Related to Figure 4. (A) U2OS cells were synchronized in mitosis with nocodazole after SCML2 knockdown, and cell-cycle progression was monitored every 2 h after release. Cell-cycle distribution for each siRNA at each time point is shown. The experiment was repeated three times, and one representative result is shown. (B) U2OS cells were transfected with a control siRNA or with siRNA #2 against SCML2. After 48 h of transfection, cells were arrested in mitosis with nocodazole for 16 h. At different times after release, cells were incubated for 5 min in the presence of EdU and the incorporation of EdU was measured by FACS using the Click-iT kit (Invitrogen).
(TIFF)

Figure S9 **Effect of SCML2 on the levels of p21 and interaction of SCML2B with CDK2/CYCE/p21-p27** ***in vivo*** **. Related to** **Figure 5** **.** (A) Western blot analysis of SCML2, p21, and USP7 in whole cell extracts from U2OS cells (control, left, or SCML2 knockdown, right) transfected with a control plasmid (Control) or a plasmid encoding SCML2A or SCML2B, as indicated. (B) Size exclusion chromatography of nuclear extract from HeLa cells using a Superose 6 column. Western blot analysis of the input (I) and the different fractions (A15 to C9) using antibodies specific for SCML2 or several cell-cycle–related proteins, as indicated on the right side.
(TIFF)

Figure S10 **Effect of the knock-down of SCML2 and p21 on their interaction with CDK2, and effect of the treatment with proteasome inhibitors on control proteins.** Related to Figure 6. (A) U2OS cells treated with siRNA control (C), siRNA #2 against SCML2 (#2), or siRNA against p21 (p21) for 48 h were arrested in mitosis with nocodazole. CDK2 was immunoprecipitated from nuclear extracts obtained at different time points after release, and the bound material was analyzed by Western blot with antibodies specific for SCML2, CDK2, and p21. For each protein the elution is shown on top and the input in the bottom. The percentage of SCML2 pulled down by CDK2 was normalized to the amount of CDK2 in the input. The percentage of pull-down in the absence of p21 was obtained compared to control-treated cells. (B) Western blot analysis of SCML2, PRSET7, and USP7, using whole cell extracts from U2OS cells treated with two different siRNAs (#1, #2) for SCML2 or a control siRNA (C) for 72 h, and then incubated in the presence of 5 µM MG132 or DMSO. (C) Immunoprecipitation of CDK2 in nuclear extracts from control-treated H9 cells (C) or cells treated with 30 µM retinoic acid for 3 and 5 d (3 d and 5 d). The material pulled-down was analyzed with specific antibodies for SCML2, CDK2, and p21. Immunoprecipitation with a nonspecific IgG is used as control, and 1% input is shown (In). (D) Western blot analysis of Nanog, p21, and p27 using nuclear extracts from control H9 cells (C), or cells treated with 30 µM retinoic acid for 3 or 5 d (3 d, 5 d). Ponceau staining is shown as loading control.
(TIFF)

Table S1 **Mass spectometry analysis of region 1 from Figure S1E.**
(DOCX)

Table S2 **Mass spectometry analysis of region 2 from Figure S1E.**
(DOCX)

Table S3 **Mass spectometry analysis of region 3 from Figure S1E.**
(DOCX)

Table S4 **Mass spectometry analysis of region 4 from Figure S1E.**
(DOCX)

Table S5 **SCML2 residues phosphorylated by CDK** ***in vitro*** **, reported phosphorylation sites and kinases predicted to act on these sites.**
(DOCX)

## Abbreviations

dRAF: dRING associated factors
DUF: domain of unknown function
ES cells: embryonic stem cells
FS: Flag-One-STrEP
MBT: malignant brain tumor
PcG: polycomb group
PhoRC: pho repressive complex
PI: propidium iodide
PRC: polycomb repressive complex
PR-DUB: polycomb repressive deubiquitinase
