# Supplementary figures and images for: Polycomb Protein SCML2 Regulates the Cell Cycle by Binding and Modulating CDK/CYCLIN/p21 Complexes
Source: PLoS Biol. 2013 Dec 17;11(12):e1001737. doi: 10.1371/journal.pbio.1001737 (PMC3866099; doi:10.1371/journal.pbio.1001737)

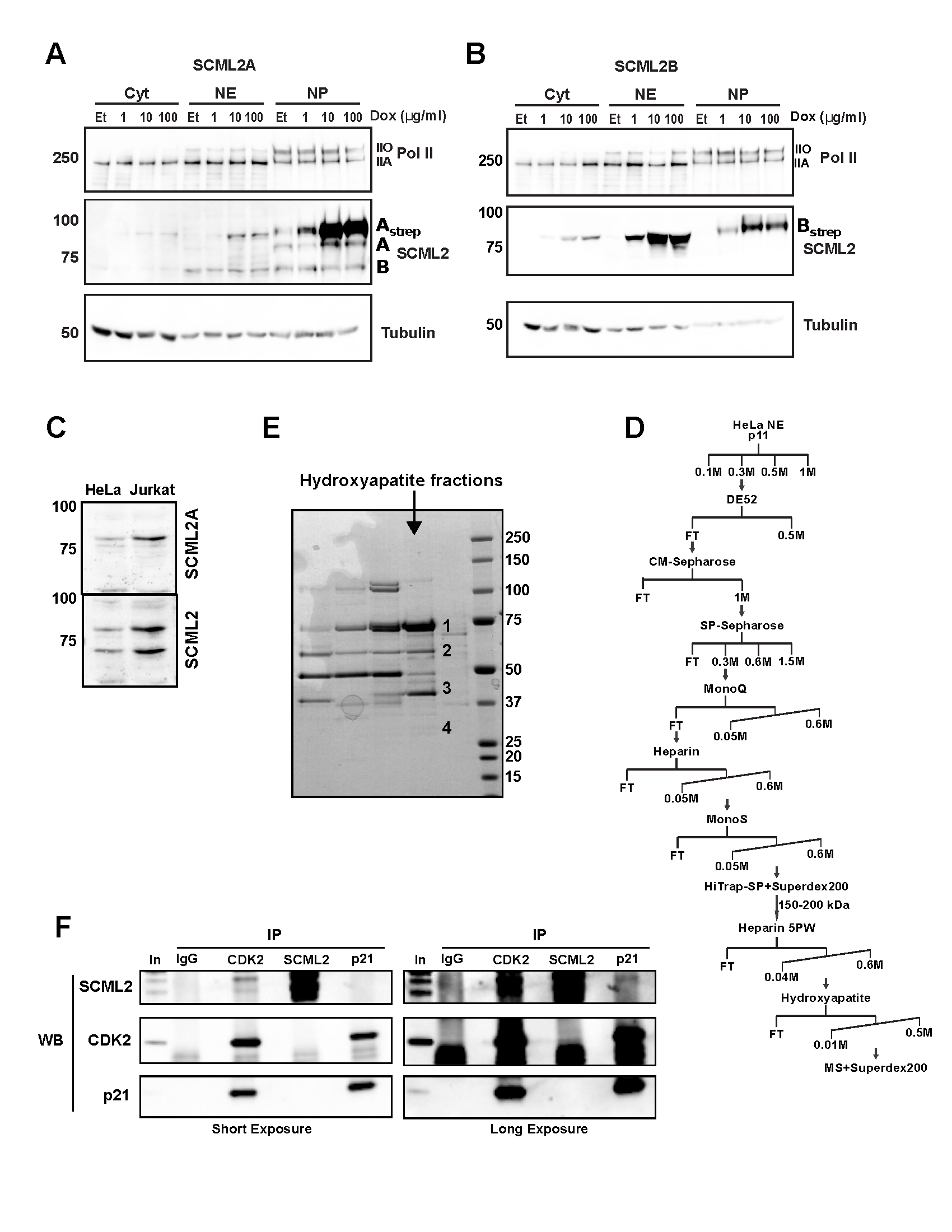

Supplement: Figure S1 — Fractionation of SCML2A and SCML2B in 293-TRex cells and purification scheme for SCML2B starting with HeLa nuclear extract. Related to Figure 1. (A–B) Stable clones of 293-TRex cells carrying an inducible plasmid for the expression of SCML2A (A) or SCML2B (B) were treated with different concentrations of doxycycline, as indicated, for 1 d. The presence of SCML2A and SCML2B in the cytosolic (Cyt), nuclear extract (NE), or nuclear pellet (NP) fractions was measured by Western blot. RNA polymerase II (O, hyperphosphorylated form; A, hypophosphorylated form) and tubulin are shown as controls for the fractionation. (C) Western blot analysis of nuclear extracts of HeLa and Jurkat cells using antibodies specific for SCML2A or recognizing both isoforms of SCML2. (D) Schematic representation of the chromatographic purification of SCML2B from HeLa nuclear extract. (E) Imperial Blue staining of the fractions from the hydroxyapatite column. The arrow indicates the peak for SCML2 elution and the numbers the regions that were cut for mass spectrometry analysis. (F) Immunoprecipitation of CDK2, SCML2 and p21 from nuclear extracts of HCT116 cells. Two percent of the input is shown along with the elution of each immunoprecipitation. A nonspecific IgG pull-down is shown as control. A short exposure of the Western blot detecting SCML2, CDK2 and p21 is shown on the left, and a log exposure on the right. (TIFF) [file pbio.1001737.s001.tiff]

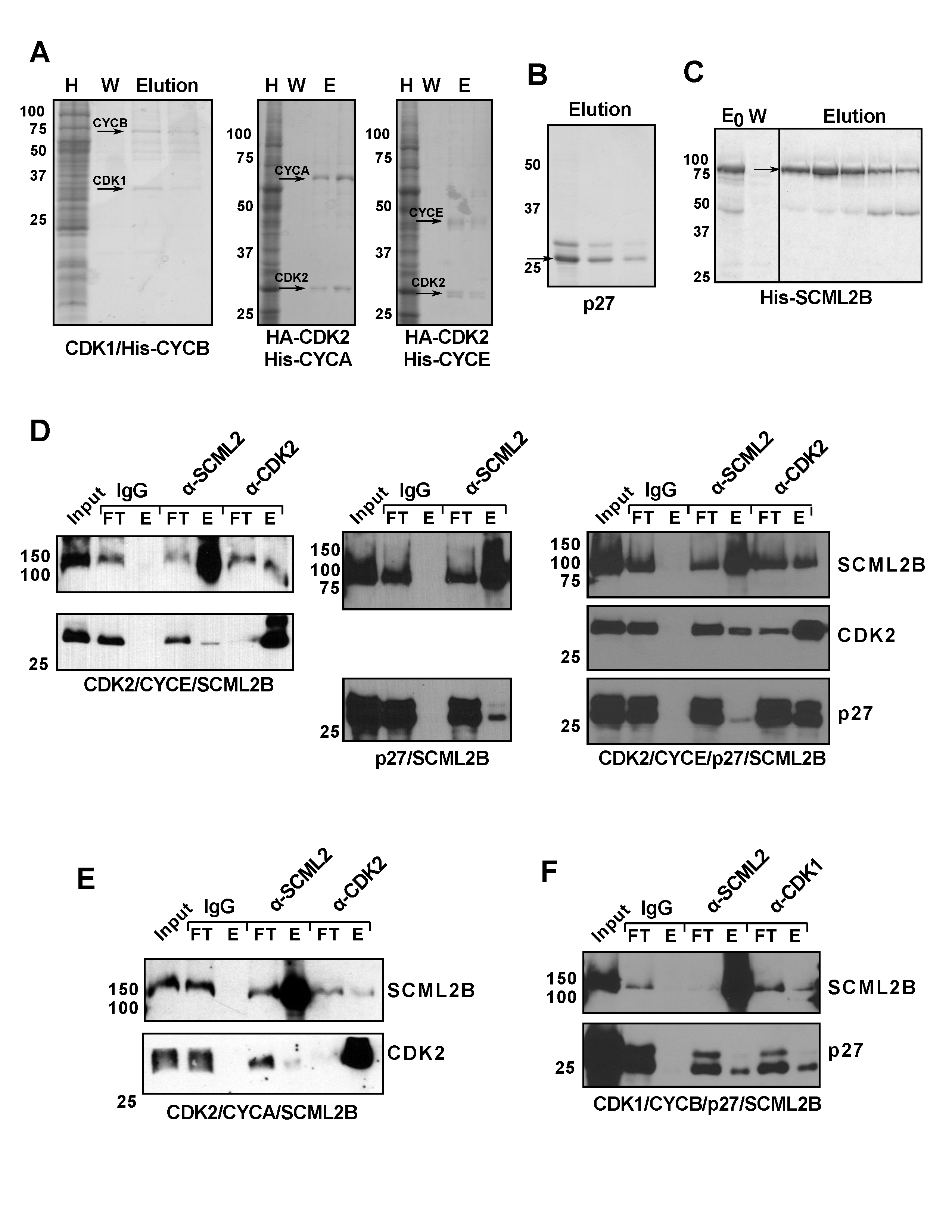

Supplement: Figure S2 — Purification of recombinant proteins and in vitro pull-down of SCML2B with CDK/CYC/p27 complexes. Related to Figure 2. (A) Sf9 cells were co-infected with baculoviruses for the different CDK/CYC combinations (kindly provided by Dr. Robert Fisher). The cells were homogenized (H) and the complexes were purified through the cyclin His tag using a Ni-NTA column. The column was washed with 30 mM imidazole (W) and the complexes were eluted with 100 and 250 mM imidazole (Elution, E). The purified complexes were dialyzed against 50 mM Tris, pH 7.5, 50 mM NaCl, and 10% glycerol. (B) GST-p27 was expressed in BL21(DE3) cells and purified using a glutathione-sepharose column. An in-column digestion was performed with the GST-prescission protease, and the digested p27 was washed away from the column (Elution). Purified p27 was dialyzed as in (A). (C) His-SCML2B was expressed in BL21(DE3) cells and purified through a Ni-NTA chromatography. After washing with 30 mM imidazole, the protein was eluted with 250 mM imidazole. This fraction (E0) was further purified in a MonoS column and eluted with a salt gradient (Elution). The fractions containing the full-length His-SCML2B were pooled and dyalyzed against 50 mM Tris, pH 7.5, 100 mM NaCl, and 10% glycerol. (D–F) CDK/CYC complexes were incubated first with p27 and then with SCML2B. The proteins were pulled down using unspecific IgG and SCML2- or CDK2-specific antibodies. Five percent of the input is shown along with the flow-through (FT) and bound (E) fractions. The pull-downs were analyzed by Western blot using specific antibodies for SCML2, CDK2, or p27, as indicated. SCML2B interaction with CDK2/CYCE, p27, and CDK2/CYCE/p27 (D), CDK2/CYCA (E), and CDK1/CYCB/p27 (F). (TIFF) [file pbio.1001737.s002.tiff]

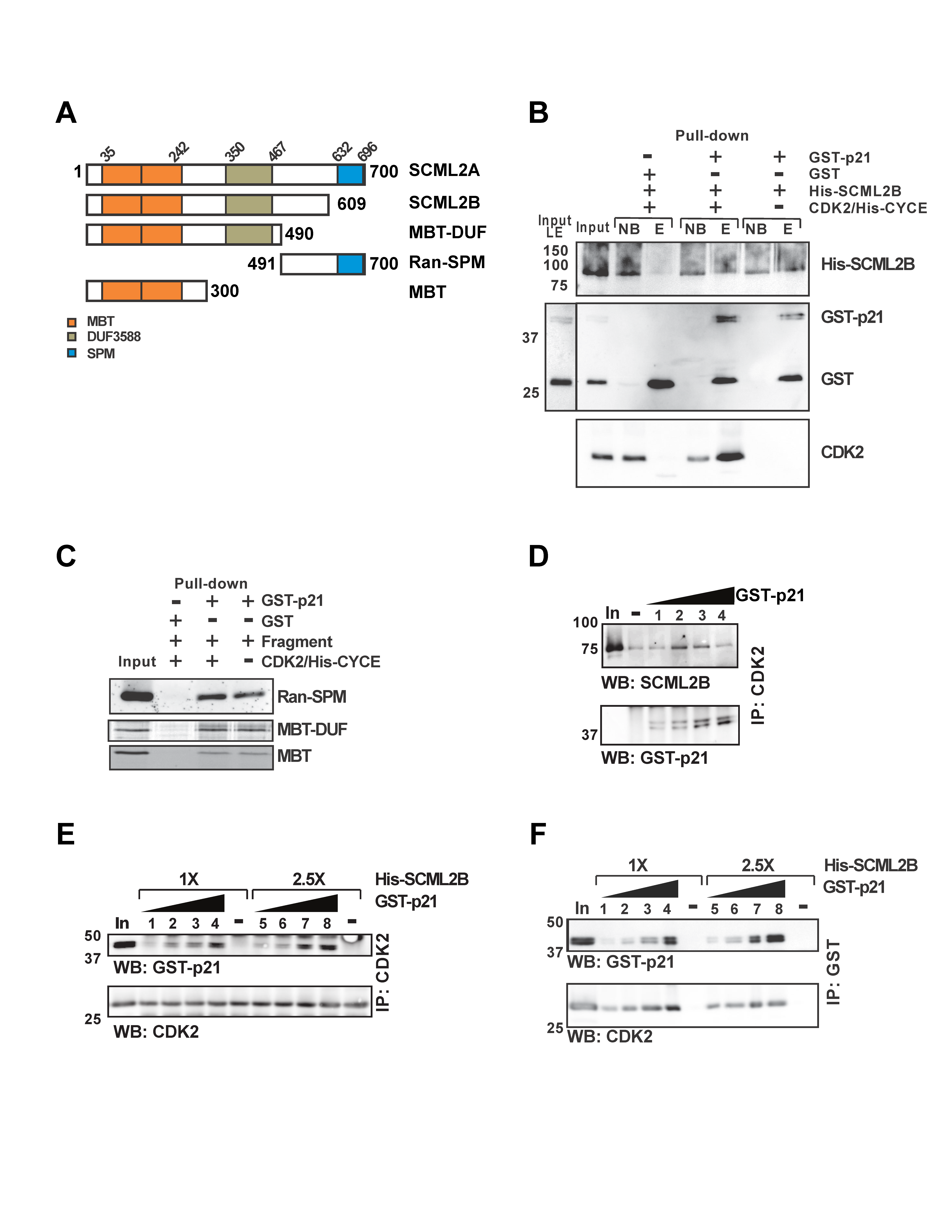

Supplement: Figure S3 — Analysis of the binding of SCML2B with CDK2/CYCE/p21. Related to Figure 2. (A) Schematic representation of SCML2A and SCML2B isoforms, together with the SCML2 fragments employed in the assays. (B) CDK2/CYCE complexes were incubated first with GST alone or GST-p21, and then with SCML2B. The proteins were pulled down using Glutathione sepharose beads. Five percent of the input is shown (a darker exposure is shown for the GST to visualize GST-p21, Input LE) along with the flow-through (FT) and bound (E) fractions. The pull-downs were analyzed by Western blot using specific antibodies for SCML2, GST, or CDK2, as indicated. (C) CDK2/CYCE complexes were incubated first with GST alone or GST-p21, and then with different fragments of SCML2B. The proteins were pulled down using Glutathione sepharose beads. Five percent of the input is shown along with the bound fractions. The pull-downs were analyzed by Western blot with antibodies for SCML2 (Ran-SPM fragment) or by silver staining (MBT-DUF and MBT fragments). (D) CDK2/CYCE complexes were first incubated with SCML2B, and then increasing amounts of GST-p21 (0.5, 1, 2, and 5× molar ratio with CDK2/CYCE) or GST alone (−) were added. Complexes were pulled down with antibodies against CDK2, and the bound material was analyzed by Western blot with antibodies specific for SCML2 or GST. Five percent of the input material (In) is shown. (E–F) CDK2/CYCE complexes were incubated with two different amounts of SCML2B, and then increasing GST-p21 or GST (−) was added. Complexes were pulled down with CDK2 antibody (E) or Sepharose-Glutathione beads (F), and the bound material was analyzed by Western blot with antibodies specific for GST or CDK2. Five percent of the input is shown (In). (TIFF) [file pbio.1001737.s003.tiff]

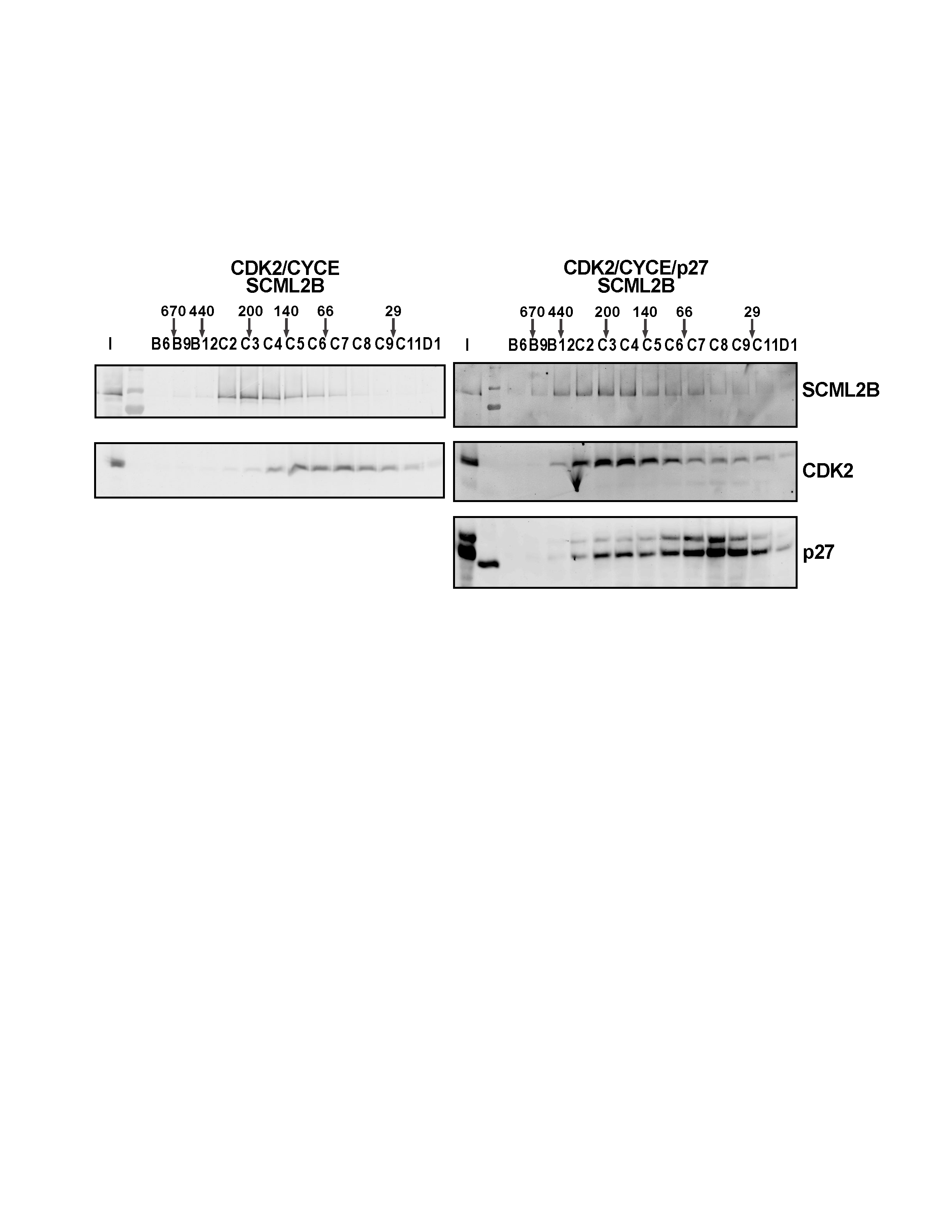

Supplement: Figure S4 — Size exclusion chromatography of CDK2/CYCE and CDK2/CYCE/p27 complexes in the presence of SCML2B. Related to Figure 2 . CDK2/CYCE complexes were incubated with p27 or SCML2B to form CDK2/CYCE/p27 and CDK2/CYCE/SCML2B complexes. Further incubation of the former complex with SCML2B yielded CDK2/CYCE/p27/SCML2B. The complexes were fractionated on Superdex 200 size exclusion chromatography. Elution profiles for the proteins indicated after fractionation of either the CDK2/CYCE/SCML2B complex (left panel), or the CDK2/CYCE/p27/SCML2B complex (right panel), as analyzed by Western blot. (TIFF) [file pbio.1001737.s004.tiff]

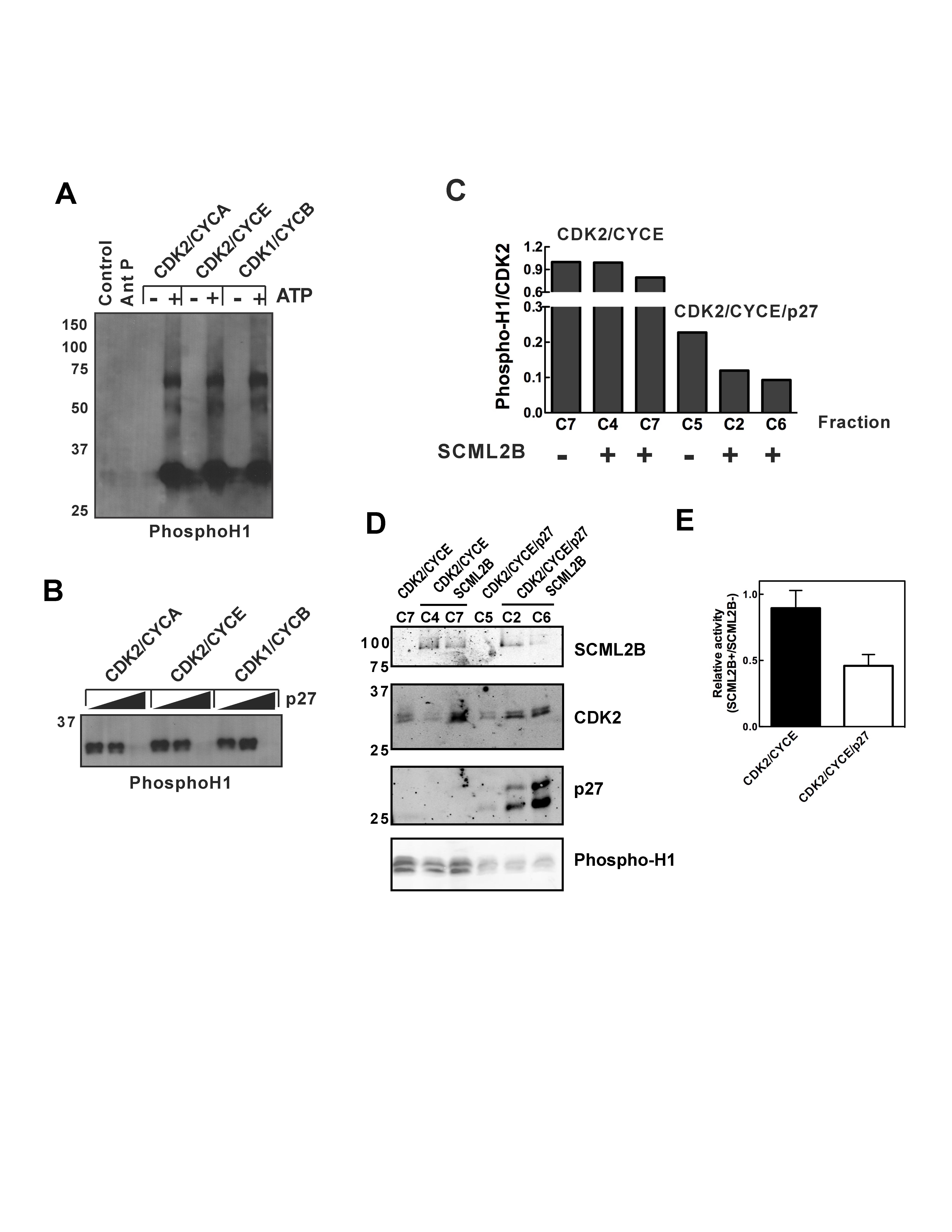

Supplement: Figure S5 — In vitro kinase assay and effect of SCML2B. Related to Figure 2. (A) CDK/CYC complexes were incubated at 30°C for 60 min with 100 ng of histone H1e in the reaction buffer, in the absence or presence of ATP. The phosphorylation of histone H1e was followed using an antibody specific for T146Phospho. The treatment of recombinant histone H1e with Antarctic phosphatase 30°C for 60 min is shown as a control; the basal phosphorylation of the substrate is negligible. (B) The kinase assay was performed as in (A) in the presence of increasing p27. (C) Kinase assays were performed employing the indicated fractions from the S200 column in Figure 3B. The levels of phosphorylated H1e (H1T146Ph) were measured by Western blot and were normalized for the levels of CDK2 in the fractions. (D) Western blot analysis of the fractions assayed in (C), with antibodies specific for SCML2, CDK2, p27, and phosphorylated H1e. (E) Relative kinase activity of fractions containing SCML2B compared to fractions without SCML2B (C) for CDK2/CYCE and CDK2/CYCE/p27 complexes. (TIFF) [file pbio.1001737.s005.tiff]

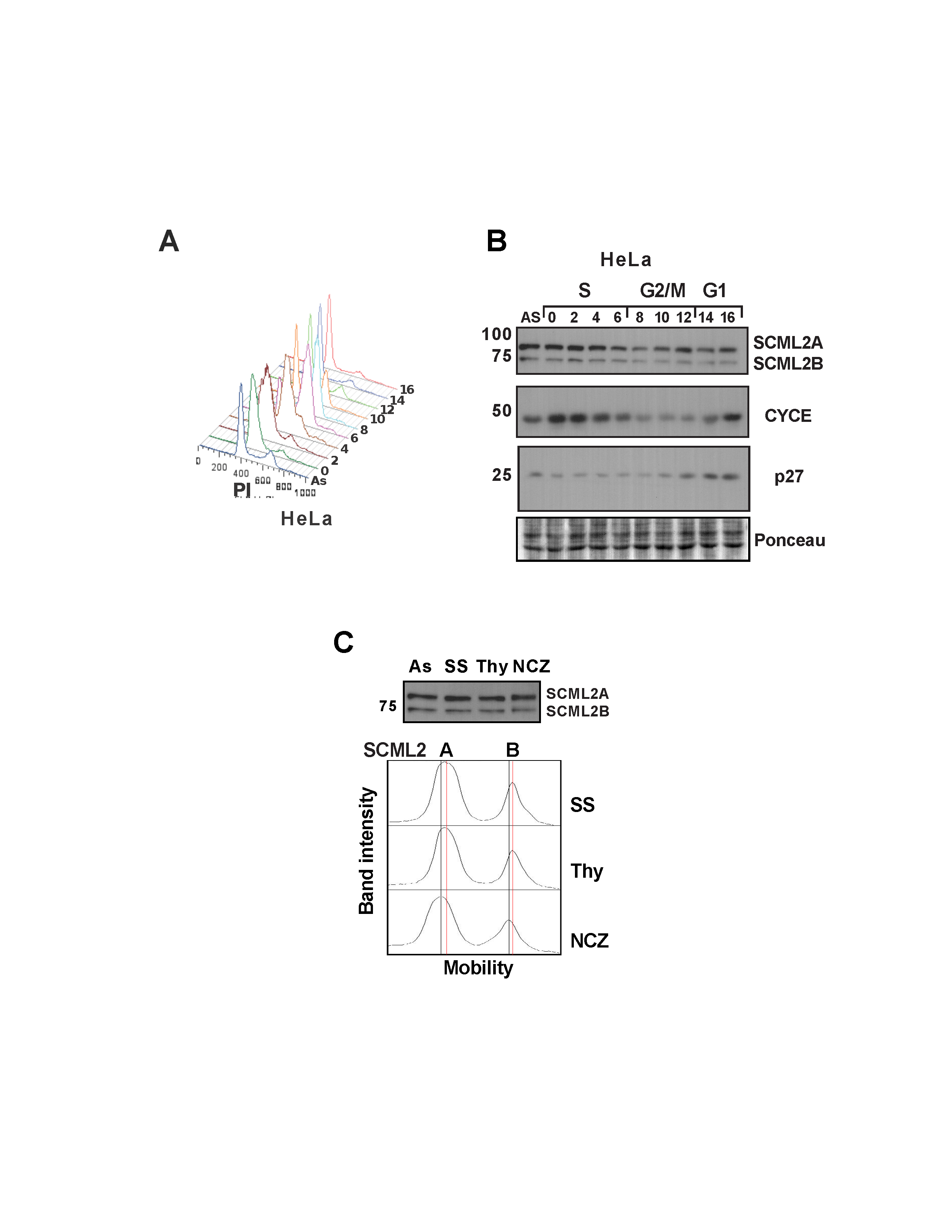

Supplement: Figure S6 — Expression and phosphorylation of SCML2 during the cell cycle. Related to Figure 3. (A) HeLa cells were synchronized at the G1/S border with a double thymidine block. Progression through the cell cycle was measured by staining with propidium iodide and analysis by flow cytommetry. (B) Western blot analysis of nuclear extracts isolated from HeLa cells every 2 h, as indicated, after cells were released from a double thymidine block. Nuclear extract from asynchronous (AS) cells is also shown. The blot was probed with antibodies specific to SCML2, CYCE2, and p27, and the membrane was stained with Ponceau Red for loading control, as indicated. (C) Nuclear extracts from HeLa cells growing asynchronously (As) or arrested in G0 (SS), G1/S (Thy), or M (NCZ) were probed for SCML2 in Western blots (upper panel). A densitometric analysis of the protein species as a function of mobility is shown (lower panel). The black line indicates the position of the peak in nocodazole-treated cells, and the red line indicates the position of the peak in thymidine-treated cells. (TIFF) [file pbio.1001737.s006.tiff]

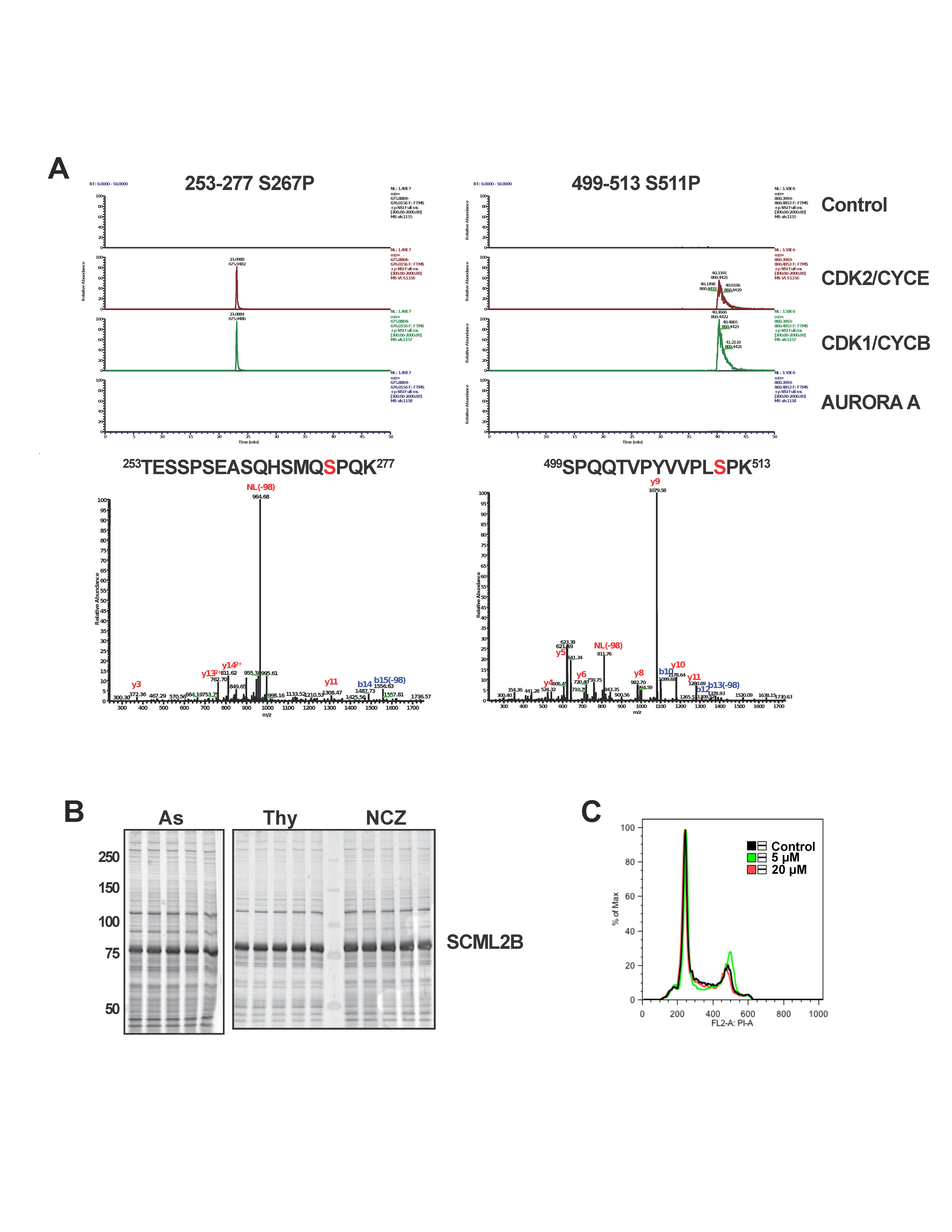

Supplement: Figure S7 — Identification of residues of SCML2B phosphorylated by CDK2/CYCE or CDK1/CYCB in vitro . Related to Figure 3 . (A) Two examples of phospho-peptides within SCML2B after treatment with CDK2/CYCE or CDK1/CYCB, identified by mass spectrometry. No signal for these peptides was detected in control or Aurora kinase A–treated SCML2B (upper panel). The position of the phosphorylation was determined by MSMS (lower panel). (B) FS-SCML2B pull-down from 293 cells growing asynchronously (As) or treated with thymidine (Thy) or nocodazole (NCZ). The same elution in each condition is loaded several times, and the bands were excised, combined, and analyzed by mass spectrometry. (C) 293T-Rex cells expressing FS-SCML2B were treated with Roscovitine for 8 h at the indicated concentrations, and the cell cycle was measured by staining with propidium iodide and analysis by flow cytommetry. (TIFF) [file pbio.1001737.s007.tiff]

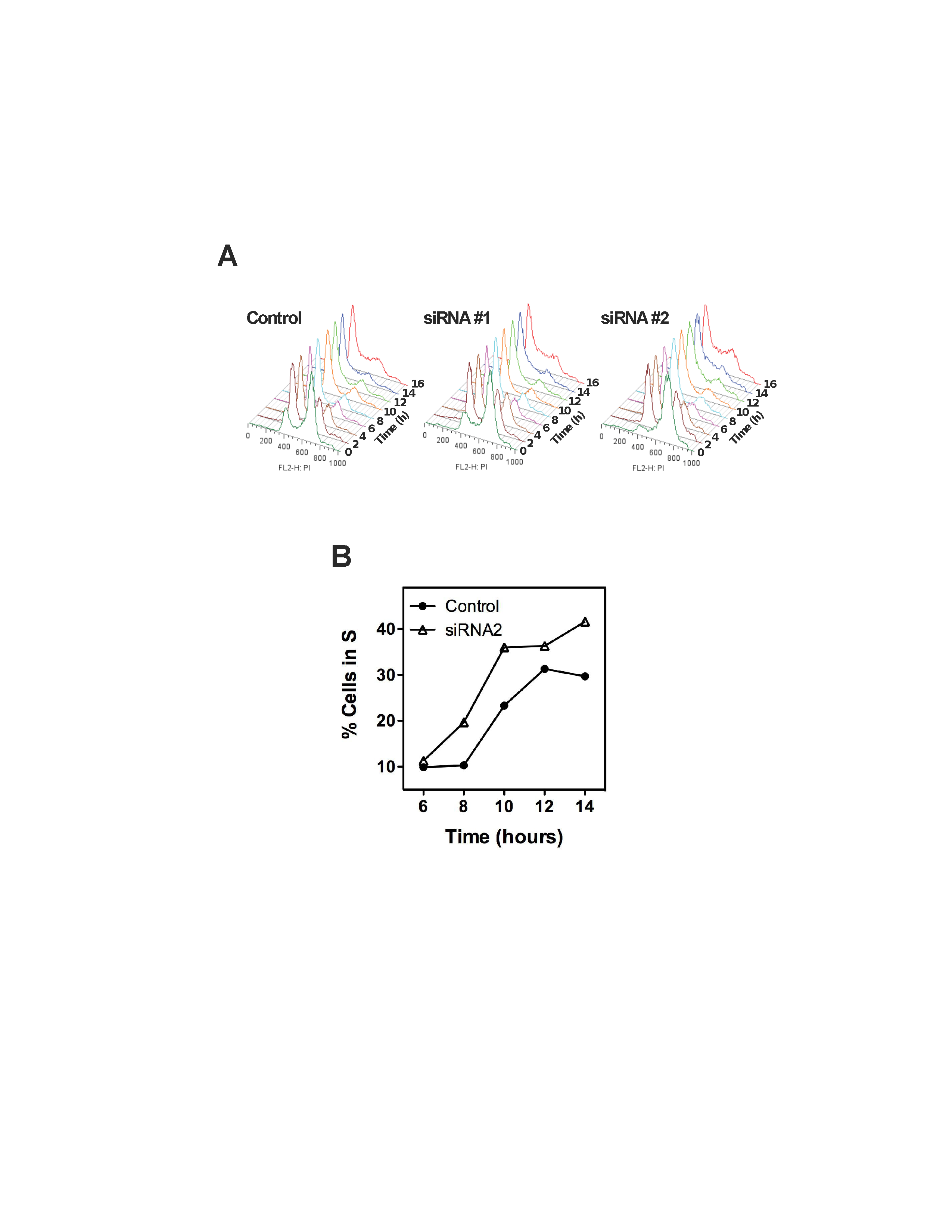

Supplement: Figure S8 — Effect of SCML2 knockdown on progression into S phase. Related to Figure 4. (A) U2OS cells were synchronized in mitosis with nocodazole after SCML2 knockdown, and cell-cycle progression was monitored every 2 h after release. Cell-cycle distribution for each siRNA at each time point is shown. The experiment was repeated three times, and one representative result is shown. (B) U2OS cells were transfected with a control siRNA or with siRNA #2 against SCML2. After 48 h of transfection, cells were arrested in mitosis with nocodazole for 16 h. At different times after release, cells were incubated for 5 min in the presence of EdU and the incorporation of EdU was measured by FACS using the Click-iT kit (Invitrogen). (TIFF) [file pbio.1001737.s008.tiff]

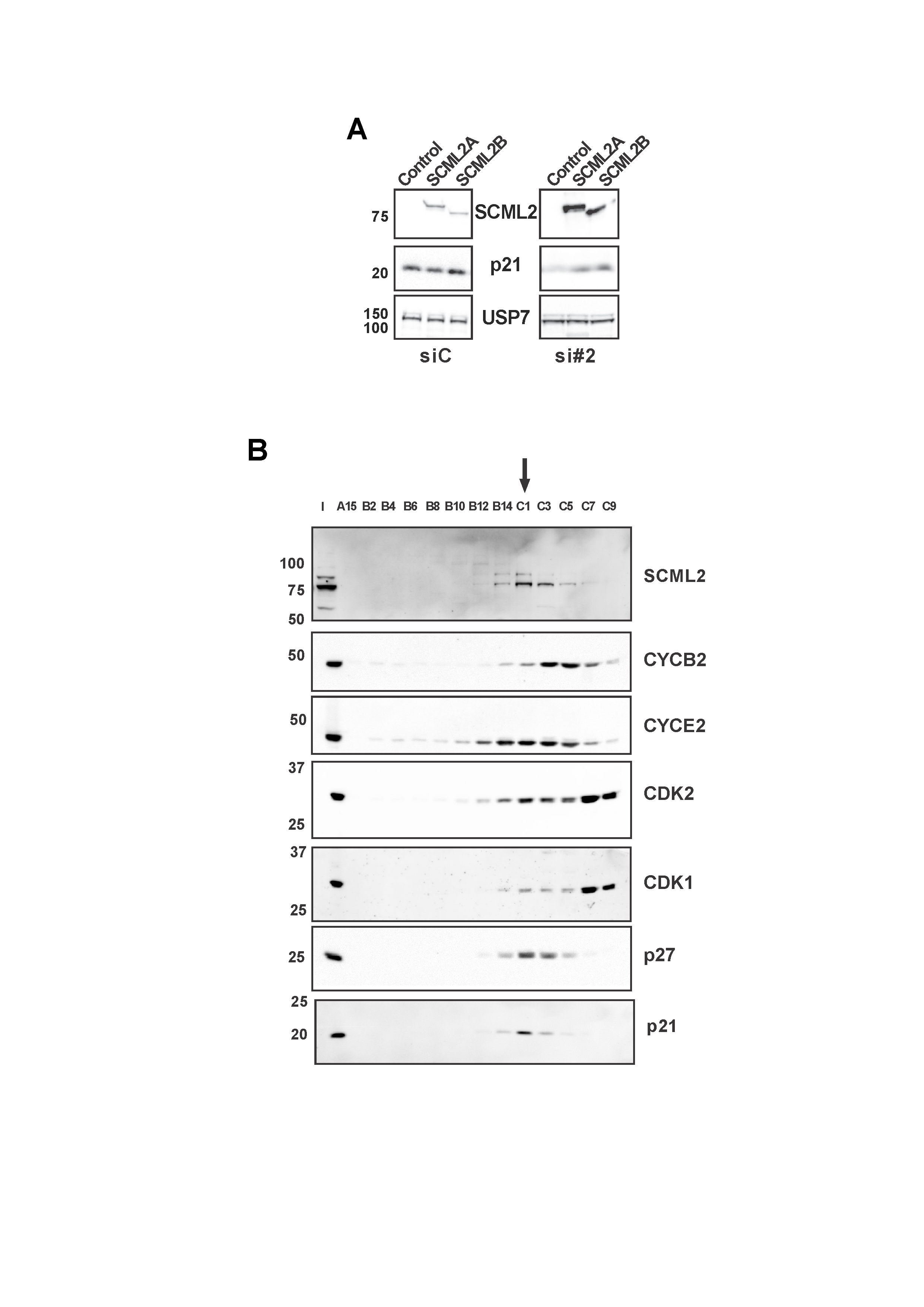

Supplement: Figure S9 — Effect of SCML2 on the levels of p21 and interaction of SCML2B with CDK2/CYCE/p21-p27 in vivo . Related to Figure 5 . (A) Western blot analysis of SCML2, p21, and USP7 in whole cell extracts from U2OS cells (control, left, or SCML2 knockdown, right) transfected with a control plasmid (Control) or a plasmid encoding SCML2A or SCML2B, as indicated. (B) Size exclusion chromatography of nuclear extract from HeLa cells using a Superose 6 column. Western blot analysis of the input (I) and the different fractions (A15 to C9) using antibodies specific for SCML2 or several cell-cycle–related proteins, as indicated on the right side. (TIFF) [file pbio.1001737.s009.tiff]

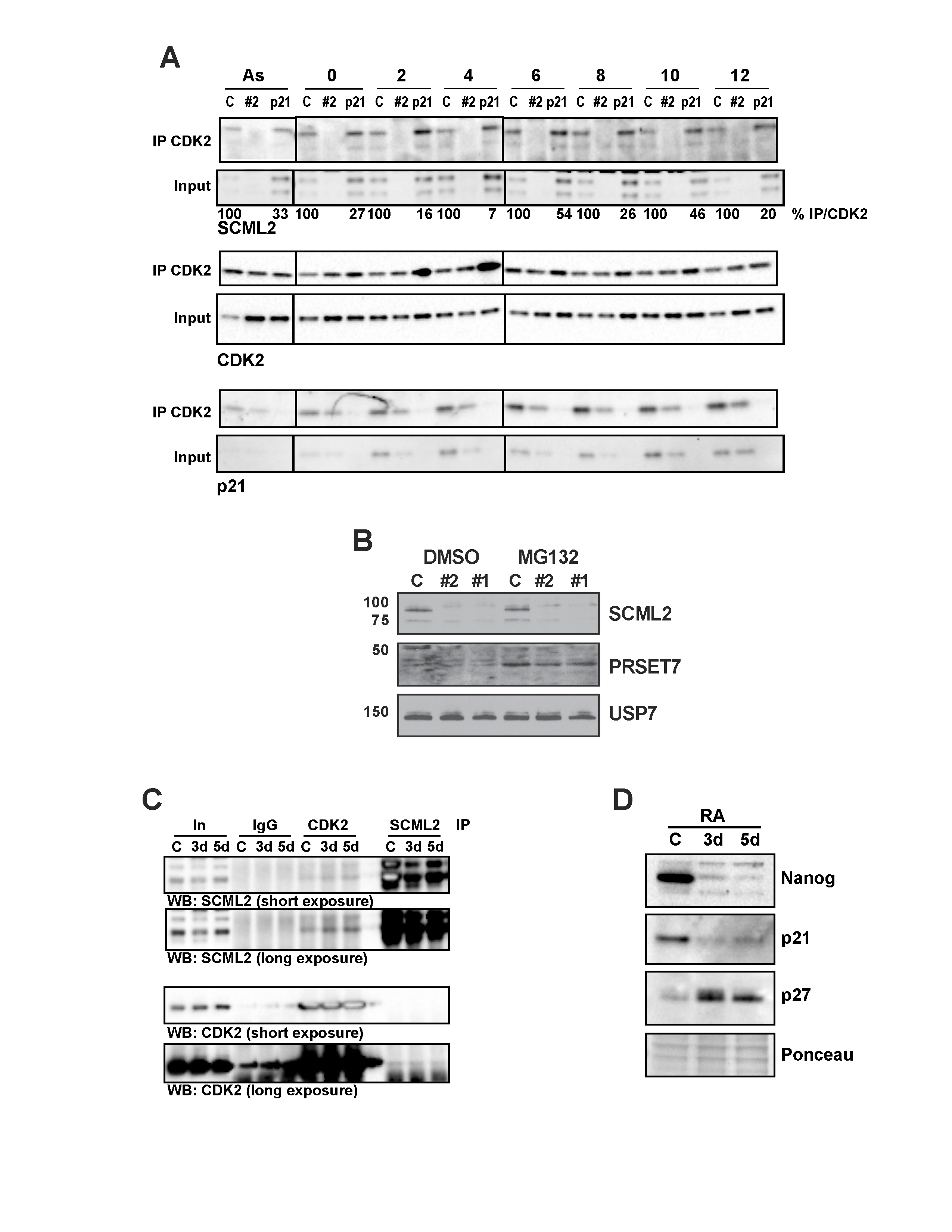

Supplement: Figure S10 — Effect of the knock-down of SCML2 and p21 on their interaction with CDK2, and effect of the treatment with proteasome inhibitors on control proteins. Related to Figure 6. (A) U2OS cells treated with siRNA control (C), siRNA #2 against SCML2 (#2), or siRNA against p21 (p21) for 48 h were arrested in mitosis with nocodazole. CDK2 was immunoprecipitated from nuclear extracts obtained at different time points after release, and the bound material was analyzed by Western blot with antibodies specific for SCML2, CDK2, and p21. For each protein the elution is shown on top and the input in the bottom. The percentage of SCML2 pulled down by CDK2 was normalized to the amount of CDK2 in the input. The percentage of pull-down in the absence of p21 was obtained compared to control-treated cells. (B) Western blot analysis of SCML2, PRSET7, and USP7, using whole cell extracts from U2OS cells treated with two different siRNAs (#1, #2) for SCML2 or a control siRNA (C) for 72 h, and then incubated in the presence of 5 µM MG132 or DMSO. (C) Immunoprecipitation of CDK2 in nuclear extracts from control-treated H9 cells (C) or cells treated with 30 µM retinoic acid for 3 and 5 d (3 d and 5 d). The material pulled-down was analyzed with specific antibodies for SCML2, CDK2, and p21. Immunoprecipitation with a nonspecific IgG is used as control, and 1% input is shown (In). (D) Western blot analysis of Nanog, p21, and p27 using nuclear extracts from control H9 cells (C), or cells treated with 30 µM retinoic acid for 3 or 5 d (3 d, 5 d). Ponceau staining is shown as loading control. (TIFF) [file pbio.1001737.s010.tiff]
