# Supplementary material for: Polycomb Protein SCML2 Regulates the Cell Cycle by Binding and Modulating CDK/CYCLIN/p21 Complexes
Source: PLoS Biol. 2013 Dec 17;11(12):e1001737. doi: 10.1371/journal.pbio.1001737 (PMC3866099; doi:10.1371/journal.pbio.1001737)
Supplement: Table S1 — Mass spectometry analysis of region 1 from Figure S1E. (DOCX) [file pbio.1001737.s011.docx]

**Table S1. Mass Spectometry analysis of region #1 from Figure S1E.**

| Protein | % Coverage | # Peptides |
| --- | --- | --- |
| **SCML2** | 66 | 890 |
| Septin-9 | 17 | 15 |
| KRT10 | 12 | 7 |
| KRT1 | 9.5 | 8 |
| KRT6B | 2.0 | 1 |
