# Supplementary material for: Polycomb Protein SCML2 Regulates the Cell Cycle by Binding and Modulating CDK/CYCLIN/p21 Complexes
Source: PLoS Biol. 2013 Dec 17;11(12):e1001737. doi: 10.1371/journal.pbio.1001737 (PMC3866099; doi:10.1371/journal.pbio.1001737)
Supplement: Table S2 — Mass spectometry analysis of region 2 from Figure S1E. (DOCX) [file pbio.1001737.s012.docx]

**Table S2. Mass Spectometry analysis of region #2 from Figure S1E.**

| Protein | % Coverage | # Peptides |
| --- | --- | --- |
| SPAST | 48 | 328 |
| KRT1 | 33 | 43 |
| KRT10 | 23 | 26 |
| **SCML2** | 23 | 20 |
| KRT2 | 18 | 11 |
| TPX2 | 14 | 14 |
| KRT9 | 17 | 11 |
| **DDX17** | 9.2 | 8 |
| KRT5 | 6.4 | 5 |
| KRT6B | 1.6 | 1 |
| KRT16 | 6.8 | 3 |
| KRT14 | 2.5 | 2 |
| ANLN | 1.8 | 1 |
