# Supplementary material for: Polycomb Protein SCML2 Regulates the Cell Cycle by Binding and Modulating CDK/CYCLIN/p21 Complexes
Source: PLoS Biol. 2013 Dec 17;11(12):e1001737. doi: 10.1371/journal.pbio.1001737 (PMC3866099; doi:10.1371/journal.pbio.1001737)
Supplement: Table S3 — Mass spectometry analysis of region 3 from Figure S1E. (DOCX) [file pbio.1001737.s013.docx]

**Table S3. Mass Spectometry analysis of region #3 from Figure S1E.**

| Protein | % Coverage | # Peptides |
| --- | --- | --- |
| HNRNPD | 53 | 177 |
| KRT1 | 30 | 38 |
| KRT10 | 22 | 19 |
| TPX2 | 16 | 18 |
| KRT9 | 22 | 17 |
| PTPN2 | 28 | 21 |
| KRT2 | 9.9 | 7 |
| ZC3H11A | 14 | 10 |
| YTHDF1 | 14 | 10 |
| YTHDF3 | 7.1 | 7 |
| **CCNE2** | 15 | 13 |
| SCML2 | 12 | 11 |
| KRT6B | 3.5 | 2 |
| **CCNB2** | 16 | 7 |
| SNORA73A | 9.3 | 3 |
| SPAST | 6.2 | 3 |
| HNRNPAB | 11 | 11 |
| MKI67 | 0.8 | 2 |
| ANLN | 1.8 | 1 |
| CMAS | 5.8 | 2 |
| KRT14 | 1.7 | 1 |
