# Supplementary material for: Polycomb Protein SCML2 Regulates the Cell Cycle by Binding and Modulating CDK/CYCLIN/p21 Complexes
Source: PLoS Biol. 2013 Dec 17;11(12):e1001737. doi: 10.1371/journal.pbio.1001737 (PMC3866099; doi:10.1371/journal.pbio.1001737)
Supplement: Table S4 — Mass spectometry analysis of region 4 from Figure S1E. (DOCX) [file pbio.1001737.s014.docx]

**Table S4. Mass Spectometry analysis of region #4 from Figure S1E.**

| Protein | % Coverage | # Peptides |
| --- | --- | --- |
| KRT1 | 40 | 53 |
| KRT10 | 32 | 31 |
| KRT9 | 23 | 31 |
| KRT2 | 21 | 18 |
| **CDK2** | 38 | 30 |
| **CDKN1B** | 59 | 26 |
| CUTC | 32 | 24 |
| KRT5 | 6.6 | 5 |
| THYN1 | 33 | 9 |
| **SCML2** | 12 | 9 |
| KRT14 | 7.2 | 3 |
| HNRNPD | 12 | 4 |
| HRNR | 2.7 | 5 |
| **CDC2** | 8.8 | 2 |
| DNMT1 | 1.7 | 5 |
