# Supplementary material for: Polycomb Protein SCML2 Regulates the Cell Cycle by Binding and Modulating CDK/CYCLIN/p21 Complexes
Source: PLoS Biol. 2013 Dec 17;11(12):e1001737. doi: 10.1371/journal.pbio.1001737 (PMC3866099; doi:10.1371/journal.pbio.1001737)
Supplement: Table S5 — SCML2 residues phosphorylated by CDK in vitro , reported phosphorylation sites and kinases predicted to act on these sites. (DOCX) [file pbio.1001737.s015.docx]

**Table S5. SCML2 residues phosphorylated by CDK *in vitro*, reported phosphorylation sites and kinases predicted to act on these sites.**

| **Phosphorylation site** | **Dephoure *et al*.** | **Olsen *et al*.** | **Kinase** |
| --- | --- | --- | --- |
| S267 | Y | N | CK1, CDK2, CDK1 |
| T277 | N | N | DDR |
| T305 | Y/Mitosis | Y | CDK2, CDK1 |
| S313 | N | N |  |
| S347 | N | N |  |
| S485 | N | N | CK2 |
| S495 | Y | N | PKA, CK2, GSK3 |
| S499 | Y/Mitosis | Y/Mitosis | CK1 |
| S511 | Y/Mitosis | Y/S | ERK, CDK1 |
| S582 | N | Y/Mitosis |  |
| S583 | Y/Mitosis | Y/Mitosis |  |
| S590 | Y/Mitosis | Y/Mitosis | GSK3, CDK1 |
| S593 | N | Y/Mitosis | CK1 |
| S594 | Y/Mitosis | Y/Mitosis | CK1, CK2 |
